# Tetrachloroethene respiration in *Sulfurospirillum* species is regulated by a two‐component system as unraveled by comparative genomics, transcriptomics, and regulator binding studies

**DOI:** 10.1002/mbo3.1138

**Published:** 2020-11-26

**Authors:** Jens Esken, Tobias Goris, Jennifer Gadkari, Thorsten Bischler, Konrad U. Förstner, Cynthia M. Sharma, Gabriele Diekert, Torsten Schubert

**Affiliations:** ^1^ Department of Applied and Ecological Microbiology Institute of Microbiology Friedrich Schiller University Jena Germany; ^2^ Leibniz Institute for Natural Product Research and Infection Biology ‐ Hans Knöll Institute Jena Germany; ^3^ Core Unit Systems Medicine University of Würzburg Würzburg Germany; ^4^ ZB MED ‐ Information Center for Life Sciences Cologne Germany; ^5^ TH Köln ‐ University of Applied Sciences Institute of Information Science Cologne Germany; ^6^ Research Group Anaerobic Microbiology Institute of Microbiology Friedrich Schiller University Jena Germany; ^7^ Present address: German Institute of Human Nutrition (DIfE) Potsdam‐Rehbrücke Nuthetal Germany; ^8^ Present address: Eurofins Umwelt Ost GmbH Jena Jena Germany

**Keywords:** genomics, organohalide respiration, RNA sequencing, tetrachloroethene, transcriptomics, two‐component system

## Abstract

Energy conservation via organohalide respiration (OHR) in dehalogenating *Sulfurospirillum* species is an inducible process. However, the gene products involved in tetrachloroethene (PCE) sensing and signal transduction have not been unambiguously identified. Here, genome sequencing of *Sulfurospirillum* strains defective in PCE respiration and comparative genomics, which included the PCE‐respiring representatives of the genus, uncovered the genetic inactivation of a two‐component system (TCS) in the OHR gene region of the natural mutants. The assumption that the TCS gene products serve as a PCE sensor that initiates gene transcription was supported by the constitutive low‐level expression of the TCS operon in fumarate‐adapted cells of *Sulfurospirillum multivorans*. Via RNA sequencing, eight transcriptional units were identified in the OHR gene region, which includes the TCS operon, the PCE reductive dehalogenase operon, the gene cluster for norcobamide biosynthesis, and putative accessory genes with unknown functions. The OmpR‐family response regulator (RR) encoded in the TCS operon was functionally characterized by promoter‐binding assays. The RR bound a *cis*‐regulatory element that contained a consensus sequence of a direct repeat (CTATW) separated by 17 bp. Its location either overlapping the −35 box or 50 bp further upstream indicated different regulatory mechanisms. Sequence variations in the regulator binding sites identified in the OHR gene region were in accordance with differences in the transcript levels of the respective gene clusters forming the PCE regulon. The results indicate the presence of a fine‐tuned regulatory network controlling PCE metabolism in dehalogenating *Sulfurospirillum* species, a group of metabolically versatile organohalide‐respiring bacteria.

## Graphical abstract

Organohalide‐respiring bacteria need to sense halogenated hydrocarbons in the environment to induce the production of the enzymatic machinery required for their utilization. Identification and characterization of biological sensors for organohalides, such as the two‐component system described here, could guide synthetic approaches for monitoring hazardous compounds at contaminated sites.

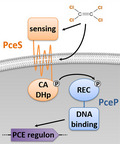

## 1 INTRODUCTION

**FIGURE 1:**
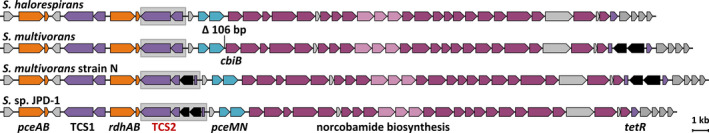
*1*Illustration of the organohalide respiration (OHR) gene region in *Sulfurospirillum* spp. The gene region encodes two reductive dehalogenases and their putative membrane anchors (orange); two two‐component systems (TCS1 and TCS2) and a TetR‐like repressor (violet); two genes that encode for components of a putative quinol dehydrogenase (turquoise, *pceMN*); and genes for the biosynthesis of the norcobamide cofactor (purple) including genes for an incomplete cobamide transport system (pink). Some of the genes have a yet unknown function (gray). Transposase genes are colored in black. *Sulfurospirillum multivorans* lacks a 106‐bp sequence in the intergenic region upstream of *cbiB*. "*Candidatus* S. diekertiae" strains SL2‐1 and SL2‐2 were not included, since the organization of their OHR gene region is identical to *Sulfurospirillum halorespirans* (Buttet et al., 2018). An overview of the locus tags is given in Table A2.

*Sulfurospirillum multivorans*, which belongs to the Campylobacterota (formerly Epsilonproteobacteria; Waite et al., 2017, 2018), gains energy from respiring chlorinated and brominated ethenes, for example, tetrachloroethene (PCE; Scholz‐Muramatsu et al., 1995; Ye et al., 2010). The terminal reductase in this organohalide respiration (OHR) is the PCE reductive dehalogenase (PceA), which was structurally analyzed before (Bommer et al., 2014; Kunze, et al., 2017). Genomic and proteomic analyses revealed the presence of a large gene region, the expression of which is induced upon cultivation with PCE as an electron acceptor (Goris, Schiffmann, et al., 2015; Goris et al., 2014). This region, which we termed the OHR gene region (Figure 1), includes *pceA* and *pceB* (encoding a putative membrane anchor) and an operon *rdhAB* for a second reductive dehalogenase. The function of the *rdhAB* is not known, especially since the expression of this operon was never observed. In addition to *pceAB* and *rdhAB*, we detected genes encoding membrane proteins probably involved in electron transfer, proteins responsible for the biosynthesis of the norcobamide cofactor of PceA, and accessory proteins putatively involved in PceA maturation (Goris et al., 2014). Regulatory proteins are also encoded in this region. Histidine kinase and response regulator genes of two‐component systems (TCSs) are located downstream of *pceAB* (TCS1) and *rdhAB* (TCS2). In general, the signaling pathway of TCSs relies on the transfer of a phosphoryl group from the sensor to the regulator protein (Stock et al., 2000). A gene for a TetR‐like regulator is positioned downstream of the norcobamide biosynthesis gene cluster. While the *tetR* gene is disrupted by a transposase in *S. multivorans*, it is intact in *Sulfurospirillum halorespirans* (Goris et al., 2017) and "*Candidatus* Sulfurospirillum diekertiae" (Buttet et al., 2018), which represent species containing an OHR gene region otherwise nearly identical to that of *S. multivorans*. The expression of *pceA* and other genes, whose products are involved in OHR, is under the control of a long‐term downregulation in *S. multivorans* (John et al., 2009). In the absence of PCE or trichloroethene (TCE), transcripts are still detectable for more than a hundred generations before the expression stops. However, as shown for cultures derived from single cells with downregulated *pceA* transcription, OHR remains inducible by PCE within one generation, excluding a loss of function (John et al., 2009). The PCE‐respiring *S. halorespirans* was shown to downregulate the OHR gene region in a similar way (Türkowsky, et al., 2018). TCS2 is conserved in both *Sulfurospirillum* species and was detected in the proteomes of OHR‐downregulated cells and thus predicted to play a role in PCE‐sensing and OHR induction. However, further evidence for the involvement of TCS2 in transcriptional regulation of the OHR gene region was missing. An acetylome study with *S. halorespirans* showed that this TCS was subject to protein acetylation (Türkowsky, et al., 2018). The molecular mechanisms responsible for the induction of OHR gene expression and the long‐term downregulation in dehalogenating *Sulfurospirillum* species are still unknown. A similar regulatory long‐term effect was not observed in other organohalide‐respiring bacteria (Kruse et al., 2016). A long‐term decrease in OHR efficiency of *Desulfitobacterium hafniense* strains cultivated in the absence of halogenated growth substrates (Futagami et al., 2006; Goris, Hornung, et al., 2015) was caused by the irreversible loss of the OHR gene cluster via transposon excision (Duret et al., 2012; Futagami et al., 2006; Maillard et al., 2005; Reinhold et al., 2012). Although the knowledge about regulatory mechanisms in organohalide‐respiring bacteria is limited, it is assumed that different bacterial genera employ different regulatory systems for the induction of OHR. Besides TCSs, the obligate organohalide‐respiring *Dehalococcoides mccartyi* harbors MarR‐type regulators (Krasper et al., 2016; Wagner et al., 2013), while the OHR gene clusters of the Firmicutes *Desulfitobacterium* and *Dehalobacter* are often encoding a CRP/FNR‐type regulator (Kruse et al., 2017; Maillard & Willemin, 2019; Türkowsky, et al., 2018) such as the functionally and structurally characterized CprK (Levy et al., 2008). In general, transcription of OHR‐related genes is non‐constitutive in versatile organohalide‐respiring bacteria (Maillard & Willemin, 2019).

Since tools for the generation of defined single‐gene knockouts in *Sulfurospirillum* spp. are not established, we used physiological experiments, comparative genomics, and gene expression studies to obtain further information on OHR in this genus (Goris et al., 2014, 2017; John et al., 2009). Further insights were derived from whole proteome analyses (Goris, Schiffmann, et al., 2015; Türkowsky, et al., 2018). Apart from *S. multivorans* and *S. halorespirans*, two other *Sulfurospirillum* species were described to be capable of OHR, *Sulfurospirillum* sp. JPD‐1 (Goris et al., 2016; Pietari, 2003) and, with a genome sequence available, "*Candidatus* S. diekertiae" (Buttet et al., 2018). Also, the non‐dechlorinating *S. multivorans* strain N was isolated from the same PCE‐dechlorinating enrichment culture that contained the dechlorinating *S. multivorans*. Interestingly, *S. multivorans* strain N was shown to contain the *pceA* gene, but no PceA protein or norcobamide cofactor has been produced by this isolate (Siebert et al., 2002).

A multilevel comparative analysis of these *Sulfurospirillum* isolates was designed to assist our efforts in uncovering the molecular basis of OHR gene regulation in PCE‐respiring *Sulfurospirillum* spp. Therefore, we combined physiological studies and genome sequencing of *S*. sp. JPD‐1 and *S. multivorans* strain N with comparative genomics and whole transcriptome analysis (RNA‐seq) of *S. multivorans* cultivated with or without PCE as the electron acceptor. Based on the results, we were able to propose a detailed transcriptional map of dehalogenating *Sulfurospirillum* spp. Besides, we identified the regulator responsible for the initiation of OHR gene transcription. DNA binding studies performed with this regulator protein uncovered its dedicated binding box and indicated the presence of a PCE regulon in dehalogenating *Sulfurospirillum* spp.

## 2 MATERIALS AND METHODS

### 2.1 Cultivation of *Sulfurospirillum* spp.

*S. multivorans* (DSM 12446^T^) and other *Sulfurospirillum* spp. were cultivated under anaerobic conditions at 28°C in a defined mineral medium (Scholz‐Muramatsu et al., 1995) without vitamin B_12_ (cyanocobalamin) and yeast extract. Pyruvate (40 mM) was used as the electron donor and fumarate (40 mM) or PCE as the electron acceptor. PCE was added to the medium (10 mM nominal concentration) from a hexadecane stock solution (0.5 M). Cultures were grown in rubber‐stoppered 200‐ml serum bottles or rubber‐stoppered 2 L Schott bottles. The ratio of aqueous to gas phase was always 1:1. To generate *S. multivorans* cells with downregulated *pceA* gene expression (John et al., 2009), the organism was cultivated for 60 transfers on pyruvate (40 mM) and fumarate (40 mM). The inoculum was 10%. The bacterial growth was photometrically monitored by measuring the optical density at 578 nm.

### 2.2 Genome sequencing, assembly, and annotation, *in silico* sequence analysis

*Sulfurospirillum multivorans* strain N (DSM 15119) was isolated earlier in the Diekert group (Siebert et al., 2002) and cultivated from a frozen glycerol stock culture, *S*. sp. JPD‐1 (DSM 16452) was received from the German Collection of Microorganisms and Cell Cultures (DSMZ). *Sulfurospirillum* sp. JPD‐1 (DSM 16452; *S. tacomaensis* BAA‐971™ in the ATCC) was isolated in the United States, close to Tacoma, Washington, as a PCE‐to‐*cis*‐dichloroethene (*c*DCE)‐dechlorinating bacterium physiologically similar to *S. multivorans* (Pietari, 2003). Genomic DNA of both organisms was isolated as reported earlier (Goris et al., 2014), and the genomes were sequenced by Macrogen, Seoul, Korea, using PacBio RSII 4.0 chemistry. The genome sequencing depth of the genomes was 180‐fold for *S. multivorans* strain N and 169‐fold for *S*. sp. JPD‐1. HGAP v3.0 was used as an assembler (with default parameters). Genomes were annotated with *S. multivorans* as reference annotation (CP72001) using Prokka (Seemann, 2014), and the annotation was manually refined. Topology prediction of membrane proteins was performed using TOPCONS (Tsirigos et al., 2015). Protein motif prediction was performed with CD‐Search (Marchler‐Bauer et al., 2017) and INTERproScan (Jones et al., 2014). The secondary structures were predicted using the Predict a Secondary Structure server (v. 6.0.0; Bellaousov et al., 2013).

### 2.3 Isolation of RNA

RNA was isolated from *S. multivorans* cells harvested after four transfers on pyruvate/PCE‐containing medium at an OD_578_ ≈ 0.26 and after 64 transfers on pyruvate and fumarate (40 mM each) at an OD_578_ ≈ 0.43. The culture (PCE‐cultivated cells: 30 ml, fumarate‐cultivated cells: 18 ml) was mixed by inversion with 1/6 volume of 95% v/v ethanol/5% v/v Roti‐Aqua‐phenol followed by 10‐min centrifugation at 1700 *g* at 4°C. The cells were snap‐frozen in liquid nitrogen and stored at −80°C.

Frozen cell pellets were thawed on ice and resuspended in 600 µl lysis solution containing 0.5 mg/ml lysozyme in TE buffer, pH 8.0, and 60 µl 10% SDS. The cells were lysed by incubating the samples for 1–2 min at 64°C. After the incubation, 1 M NaOAc, pH 5.2 (66 µl), was added and the sample was mixed by inversion. Total RNA was extracted by adding 750 µl phenol (hot‐phenol method). The solution was mixed by inversion and incubated for 6 min at 64°C. Afterward, the samples were mixed 6–10 times by inversion and cooled on ice. After centrifugation for 15 min at 14,000 *g* at 4°C, the aqueous layer was transferred and the chloroform extraction was performed in a 2‐ml Phase Lock Gel tube (Eppendorf). 750 µl chloroform was added and mixed by inversion. After centrifugation for 12 min at 14,000 *g* and 15°C, the aqueous layer was used for the ethanol precipitation. To the RNA containing the sample, 0.1 volume of 3 M NaOAc, pH 5.2, and two volumes of 96% ethanol (−20°C) were added. The sample was incubated for 2 h at −20°C. Ethanol was discarded after centrifugation for 20 min at 14,000 *g* and 4°C. The RNA was washed once with 200 µl of 70% ethanol (−20°C). Ethanol was removed, and the RNA was used for sequencing.

To prepare RNA samples for RT‐qPCR, approximately 1 × 10^9^ *S. multivorans* cells were harvested during the exponential growth phase (OD_578_ ≈ 0.15). Total RNA was isolated using the RNeasy Mini Kit (Qiagen). The DNA was digested with recombinant DNase I (RNase‐free, Roche) in the presence of RNase inhibitor (RiboLock, Thermo Scientific).

### 2.4 Reverse transcription‐PCR (RT‐PCR)

The One‐Step RT‐PCR Kit (Qiagen) was used. The reaction mixture contained 5 µl 5× reaction buffer, 25 pmol reverse primer, 25 pmol forward primer, 1 µl 10 mM dNTP mix, 1 µg total RNA or 70 ng *S. halorespirans* genomic DNA as a positive control, 1 µl enzyme mix, and nuclease‐free water up to a final volume of 25 µl. As a negative control, nuclease‐free water was added instead of the nucleic acid. The reaction mixture was incubated for 1 h at 50°C followed by a PCR with an initial denaturation of 95°C for 15 min, followed by a defined number (given in Results section) of cycles including denaturation for 1 min at 94°C, annealing for 30 s at 50°C, and elongation for 1 min at 72°C. The final elongation step lasted 10 min. The amplified DNA was separated on a 2% agarose gel and stained with ethidium bromide. The primers used for RT‐PCR analysis are listed in Table A1. The 16S cDNA was diluted 1:10.000 before PCR.

### 2.5 Reverse transcription–quantitative real‐time PCR (RT‐qPCR)

The RevertAid First‐Strand cDNA Synthesis Kit (Thermo Scientific) was used for reverse transcription. The reaction mixture contained 3.5 µl 5× reaction buffer, 25 pmol reverse primer, 2 µl 10 mM dNTP mix, 1 µg total RNA, and nuclease‐free water up to a final volume of 17.5 µl. A volume of 10 µl was transferred into a new PCR tube, and 0.5 µl reverse transcriptase was added. The remaining mixture was used as a reverse transcriptase minus (RT‐) negative control to assess for genomic DNA contamination in the RNA sample. The reaction mixtures were incubated for 1 h at 42°C plus 5 min at 70°C to stop the RT reaction. The qPCR was performed in technical triplicates using a CFX96 qPCR machine (Bio‐Rad). Each reaction mixture contained 6 µl 2× Maxima SYBR Green qPCR Master Mix (Fermentas), 5 pmol of both forward and reverse primers, and finally, either 2.5 µl cDNA sample, 175 ng *S. halorespirans* genomic DNA as a positive control, or nuclease‐free water as a negative control. The mixtures were filled up to a final volume of 12 µl with nuclease‐free water. The initial denaturation was performed for 3 min at 95°C, followed by 40 cycles of denaturation for 15 s at 95°C, annealing for 30 s at 50°C, and extension for 30 s at 72°C. Directly after qPCR, melting curves were measured and the transitions were checked for primer dimer formation and false PCR products. The primers used for RT‐qPCR analysis are listed in Table A1.

### 2.6 cDNA library preparation and sequencing

Terminator exonuclease (TEX) treatment of RNA samples was performed as previously described (Sharma et al., 2010). The cDNA libraries for Illumina sequencing were constructed by Vertis Biotechnology AG, Germany (http://www.vertis‐biotech.com/), in a strand‐specific manner as previously described for eukaryotic microRNA (Berezikov et al., 2006) but omitting the RNA size‐fractionation step before cDNA synthesis.

In brief, the RNA samples were poly(A)‐tailed using poly(A) polymerase. Terminator exonuclease treatment (+TEX) and mock treatment without the enzyme (‐TEX) were carried out after poly(A)‐tailing. In this way, corresponding cDNA pairs were generated. Then, the 5'PPP structures were removed using tobacco acid pyrophosphatase (TAP). Afterward, an RNA adapter was ligated to the 5'‐monophosphate of the RNA. First‐strand cDNA synthesis was performed using an oligo(dT)‐adapter primer and the M‐MLV reverse transcriptase. The resulting cDNAs were PCR‐amplified to about 10‐20 ng/μl using a high‐fidelity DNA polymerase. The cDNAs were purified using the Agencourt AMPure XP kit (Beckman Coulter Genomics) and were analyzed by capillary electrophoresis.

For Illumina sequencing, samples were pooled in approximately equimolar amounts. The cDNA pool was size‐fractionated in the size range of 150‐600 bp (replicate A) or 200‐600 bp (replicate B) using a differential clean‐up with the Agencourt AMPure kit. An aliquot of the cDNA pool was analyzed by capillary electrophoresis.

The primers used for PCR amplification were designed for TruSeq sequencing according to the instructions of Illumina. The following adapter sequences flank the cDNA inserts:

TruSeq_Sense_primer:

5'‐AATGATACGGCGACCACCGAGATCTACACTCTTTCCCTACACGACGCTCTTCCGATCT‐3'

TruSeq_Antisense_NNNNNN_primer Barcode:

5'‐CAAGCAGAAGACGGCATACGAGAT‐NNNNNN‐GTGACTGGAGTTCAGACGTGTGCTCTTCCGATC(dT25)‐3'. The combined length of the flanking sequences is 146 bases. All libraries were sequenced on an Illumina HiSeq 2500 machine with 100 cycles in single‐end mode.

### 2.7 Computational analysis of dRNA‐seq data

To assure high sequence quality, Illumina reads in FASTQ format were quality‐trimmed with a cutoff Phred score of 20 using fastq_quality_trimmer (FASTX‐Toolkit version 0.0.13, http://hannonlab.cshl.edu/fastx_toolkit/) discarding reads without any remaining bases. High‐quality reads were converted to FASTA format via fastq_to_fasta (FASTX‐Toolkit). Afterward, we applied the pipeline READemption (Förstner et al., 2014) version 0.3.5 for trimming of poly(A)‐tail sequences and to align all reads longer than 11 nt to the *Sulfurospirillum multivorans* DSM 12446^T^ (GenBank Acc.‐No.: CP007201.1) genome using segemehl version 0.2.0 (Hoffmann et al., 2009) with an accuracy cutoff of 95%. To facilitate visualization in a genome browser, coverage plots representing the numbers of mapped reads per nucleotide were generated. Reads that mapped to multiple locations contributed a fraction to the coverage value. For example, reads mapping to three positions contributed only 1/3 to the coverage values. Each graph was normalized to the number of reads that could be mapped from the respective library. To restore the original data range, each graph was then multiplied by the minimum number of mapped reads calculated over all libraries.

We applied READemption to assess the overlap of read alignments for each library to GenBank annotations for CDS, tRNA, rRNA, and ncRNA features (CP007201.1, download on 2015‐09‐22) on the sense strand. Each read with a minimum overlap of 10 nt was counted with a value based on the number of locations where the read was mapped. If the read overlapped more than one annotation, the value was divided by the number of regions and counted separately for each region (e.g., 1/3 for a read mapped to three locations). The resulting read counts were subjected to differential expression analysis of Py_PCE versus Py_Fu total RNA samples (‐TEX) using DESeq2 (Love et al., 2014) version 1.8.1 via READemption. All features with log2FoldChange ≤−1 or ≥1 and Benjamini–Hochberg‐corrected *p*‐values (*p*adj) <0.05 were considered significantly differentially expressed. The Integrated Genome Browser (Freese et al., 2016) was used for the dRNA‐seq data evaluation.

### 2.8 Heterologous production of the response regulator protein

The gene encoding PceP (SHALO_1503) was amplified from *S. halorespirans* genomic DNA using the cloning primers listed in Table A2, digested with the restriction enzyme Esp3I, and ligated to the multiple cloning site of pASG‐IBA105 (IBA). The construct was verified by sequencing using the sequencing primers listed in Table A2. *E. coli* DH5α was used for plasmid proliferation. *E. coli* BL21(DE3) was used for PceP‐twin‐Strep overproduction. Both strains were cultivated in lysogeny broth (LB) containing 100 µg/ml ampicillin. For gene expression, the cultivation temperature was set to 28°C and reduced to 18°C after induction with 100 µg/L anhydrotetracycline. The cells were harvested after 1 h.

### 2.9 Electrophoretic mobility shift assay (EMSA)

Twin‐Strep‐tagged PceP protein was purified on Strep‐Tactin XT Superflow columns (IBA) using the standard protocol provided by the manufacturer. The purity was proven by Coomassie‐ or silver‐stained polyacrylamide gels. Silver staining comprised fixation for 20 min (50% (v/v) methanol, 10% (v/v) acetic acid, 10 mM ammonium acetate), followed by two washing steps for 10 min with ultra‐pure water, sensitization for 20 min (20 µM sodium thiosulfate), staining for another 20 min (0.6 mM silver nitrate), washing with ultra‐pure water, developing (0.1% (v/v) formaldehyde, 20 mM sodium carbonate) for about 1 min, and finally stopping for 10 min (50 mM EDTA).

The 6FAM‐labeled double‐stranded DNA probes contain the entire promoters or truncated sequences and were amplified from *S. halorespirans* genomic DNA using the indicated primers (Table A3). EMSA reactions (15 µl) contained 2.5 pmol DNA probe, 250 ng (≈0.1 pmol) poly (dI/dC), and the indicated amounts of PceP‐twin‐Strep in 1× reaction buffer (10 mM HEPES, pH 7.6, 2 mM MgCl_2_ ∙ 6 H_2_O, 200 mM KCl, 0.1 mM EDTA, 5 mM DTT, 10% (v/v) glycerol, 50 µg/µl Ficoll 70). The reaction was performed at room temperature for 20 min. The reaction mixtures were separated using a protocol adapted from Sidorova et al. (2010). The native 10% polyacrylamide gels (acrylamide:bis‐acrylamide.=29:1) contained 30% (v/v) triethylene glycol in 1× TAE buffer (40 mM Tris, pH 8.3, 20 mM acetic acid, 2 mM EDTA). 20 µl of 30% (v/v) triethylene glycol in 1× TAE was added to the sample wells as soon as the gel was immersed in the electrophoresis buffer to minimize the loss of the osmolyte. The gels were run at 200 V for 2 h and were scanned with a Typhoon FLA 7000 device (GE Healthcare) with a 473 nm LD laser and a Y520 filter.

## 3 RESULTS

### 3.1 The genomes of *Sulfurospirillum multivorans* strain N and *S*. sp. JPD‐1

**FIGURE A1:**
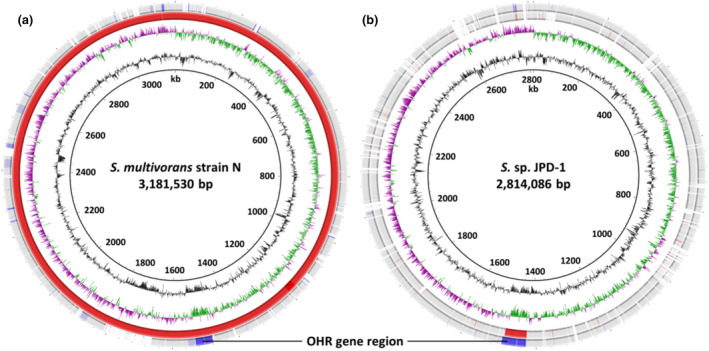
Circular representations of the genomes of A) *S*. *multivorans* strain N (acc. no.: CP042699) and B) *S*. sp. JPD1 (acc. no.: CP023275) aligned with those of *S*. *halorespirans* (outer circle; acc. no.: CP017111.1) and *S*. *multivorans* (second circle from outside; acc. no.: CP007201.1). Blast hits with more than 99% nucleotide identity are colored in blue or red, respectively, and hits with 95–99% identity are shown in a pale color, respectively. The third circle shows the GC skew (green and purple), and the fourth circle shows the GC content (black). Positions are given in the inner circle. The images were generated with BRIG (Alikhan et al., 2011).

In a search for the genetic defect causing the inability of *S. multivorans* strain N to reductively dechlorinate PCE, we sequenced its genome (Table A4; Figure A1a). The genome of strain N (DSM No.: 15119) is nearly identical (average nucleotide identity of 99.99% as calculated with ANI calculator; Goris et al., 2007) to that of the dechlorinating *S. multivorans* (DSM No.: 12446^T^). The most apparent difference between the two strains is the location of transposase genes, which are numerous in the genome of *S. multivorans* (Goris et al., 2014). The OHR gene region of both *S. multivorans* strains is identical with two exceptions. In strain N, a transposase gene (SMN_1525) disrupts the gene encoding the response regulator (RR) of TCS2 (SMN_1524) and a short stretch (106 bp) of an additional DNA sequence is present in the intergenic region upstream of the first gene (*cbiB*) of the norcobamide biosynthesis gene cluster (Figure 1). These 106‐bp sequences have been detected before in the OHR gene regions of *S. halorespirans* and "*Candidatus* S. diekertiae" strains, too (Buttet et al., 2018; Goris et al., 2017).

**FIGURE A2:**
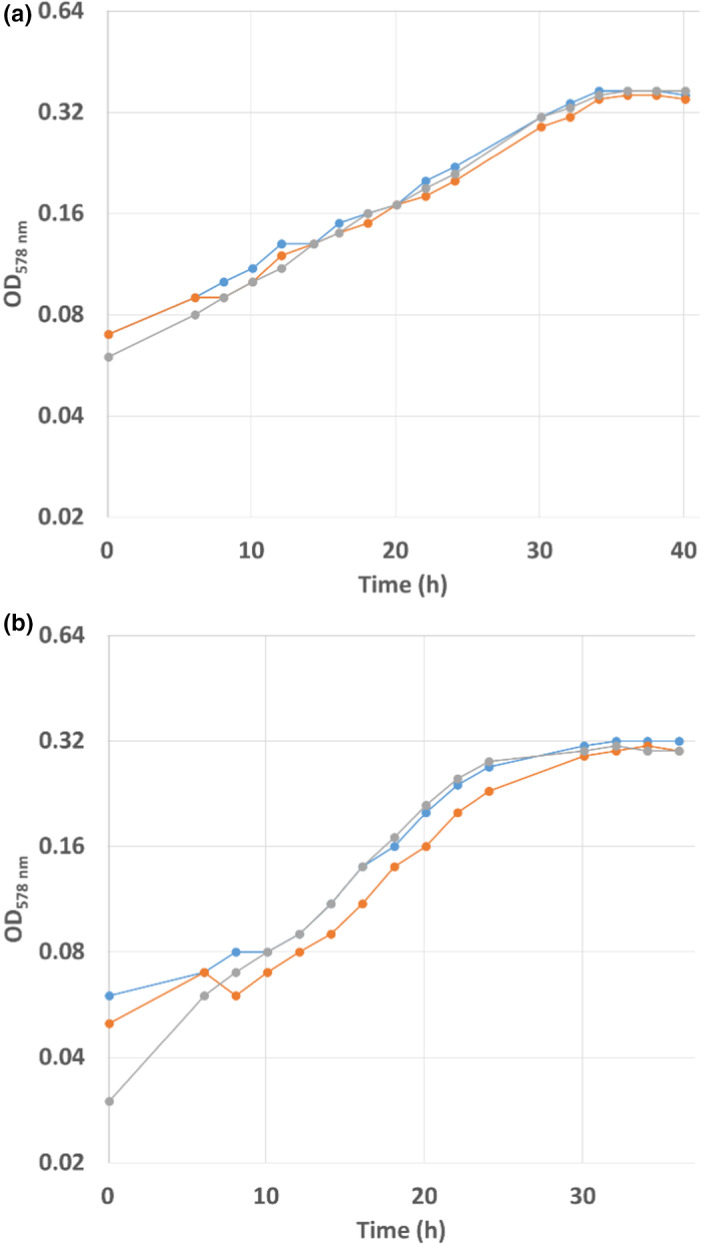
Growth curves of (a) *S*. sp. JPD‐1 and (b) *S*. *multivorans* strain N on pyruvate and fumarate (40 mM each). Three biological replicates were measured.

Opposed to its original description (Pietari, 2003), *S*. sp. JPD‐1 was not able to dechlorinate PCE or TCE regardless of the electron donor (formate or pyruvate), and the presence or absence of vitamin B_12_ or yeast extract in the medium. Fumarate served as the alternative electron acceptor (Figure A2). To determine the reason for its inability to dechlorinate PCE, the genome of the species was sequenced (Table A4; Figure A1b). Similar to the results obtained for *S. multivorans* strain N, a transposase, here comprising two genes, disrupted the RR gene of TCS2 of *S*. sp. JPD‐1. The total number of transposases in JPD‐1 is much lower compared with *S. multivorans*. A conserved domain search revealed only 16 transposase genes in *S*. sp. JPD‐1, while *S. multivorans* and its non‐dechlorinating counterpart strain N harbor about 80 genes related to transposable elements such as transposases, integrases, and phage‐dependent recombinases. The 106‐bp additional DNA sequence in the intergenic region upstream of the *cbiB* gene is also present in *S*. sp. JPD‐1.

The OHR gene region is highly conserved with nearly 100% sequence identity in all *Sulfurospirillum* species and strains containing this region. The sequence identity exceeds those of the genes for ribosomal RNAs and proteins (98‐99%). The OHR gene region contains 44 genes plus transposable elements and was described before in detail for *S. multivorans* (Goris et al., 2014) and *S. halorespirans* (Goris et al., 2017; Figure 1). In comparison with all other genes in the OHR gene region, the *pceA* gene displays the lowest sequence identity among all the species analyzed here (approximately 95%). In contrast, the product of the second reductive dehalogenase gene *rdhA* is identical in all isolates. Neither a *rdhA* transcript in *S. multivorans* (Goris et al., 2014) nor the RdhA protein in *S. multivorans* (Goris, et al., 2015) or *S. halorespirans* (Türkowsky, et al., 2018) were detected in previous studies.

The transposase genes disrupting the gene encoding the response regulator of TCS2 (RR2) in *S. multivorans* strain N and *S*. sp. JPD‐1 are located at different positions in the coding sequence and are not phylogenetically related to each other. The transposase gene disrupting the RR2 gene in *S. multivorans* strain N appeared in several copies in the genomes of *S. multivorans*, *S. halorespirans*, and strain N itself (Table A6). The transposable element disrupting RR2 in *S*. sp. JPD‐1 (SJPD1_1513 and SJPD1_1514) was also identified at other loci in JPD‐1, *S. multivorans*, *S. multivorans* strain N, and "*Candidatus* S. diekertiae." The TetR‐like regulator‐encoded downstream of the norcobamide biosynthesis gene cluster is intact in *S*. sp. JPD‐1 and *S. halorespirans*, while it is disrupted by the same transposable element in both *S. multivorans* strains (Figure 1).

### 3.2 Transcriptome of *S. multivorans* cultivated with PCE or fumarate

**FIGURE 2:**
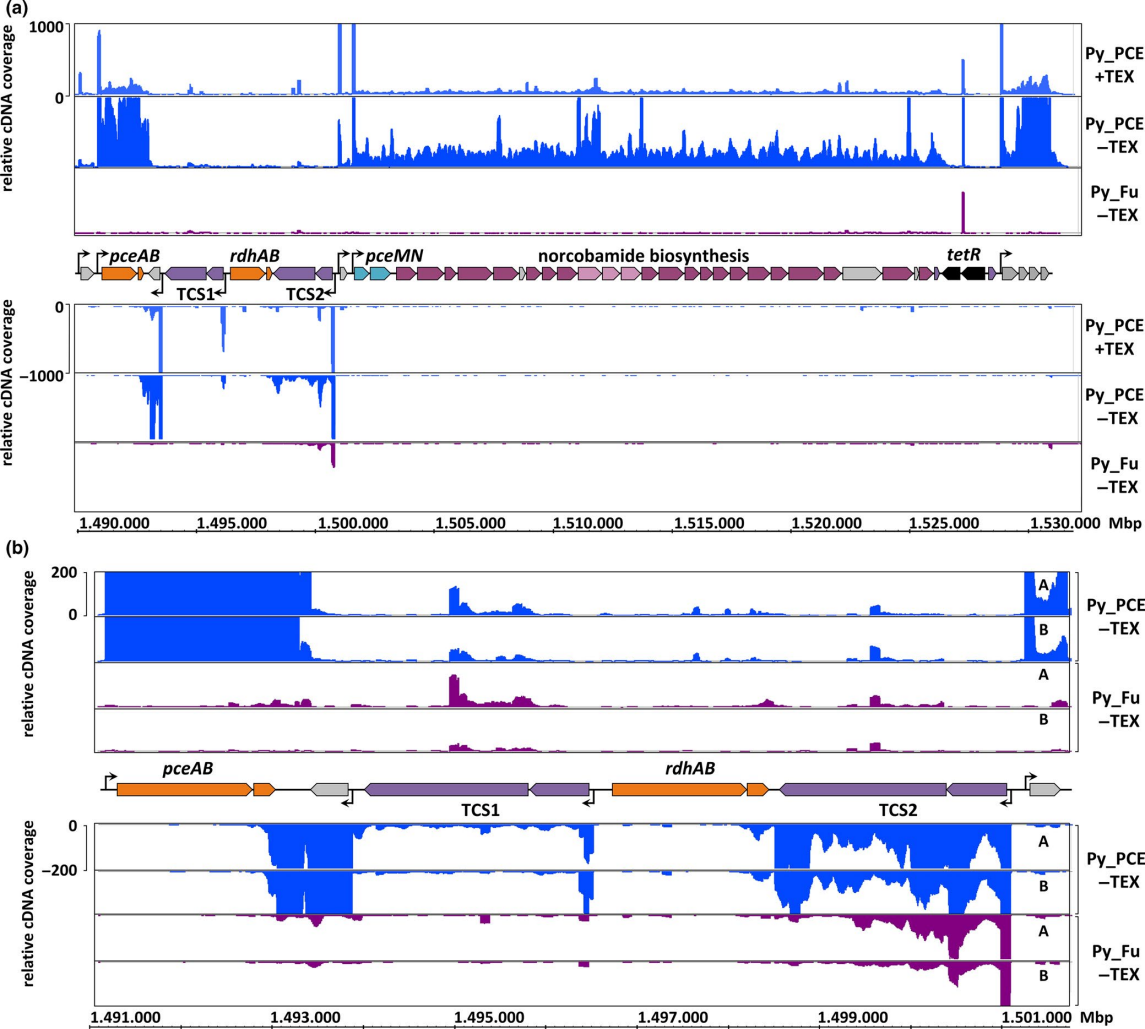
Differential RNA sequencing (dRNA‐Seq) results of the *Sulfurospirillum multivorans* OHR gene region. (a) Data for the complete OHR gene region. Only replicate B is shown. The analysis led to the annotation of global transcriptional start sites (TSS) marked by arrows. The “Integrated Genome Browser” (v. 9.0.1) was used for data evaluation (Freese et al., 2016). For the read alignment statistics, please refer to Table A7. (b) RNA sequencing results focusing on the genes encoding the reductive dehalogenases and the two TCSs in the OHR gene region. Both biological replicates (A and B) are displayed.

**FIGURE A3:**
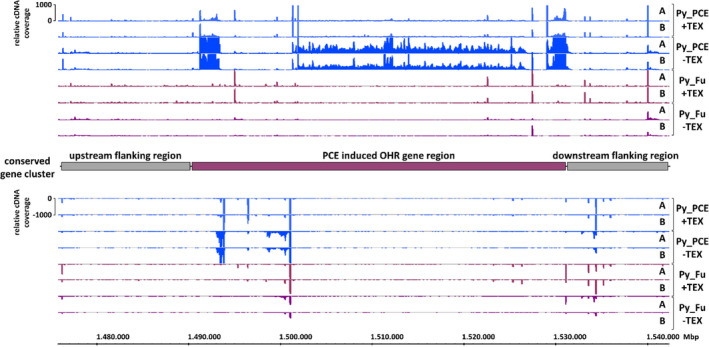
Differential RNA sequencing (dRNA‐Seq) results of the gene region that is conserved among the *Sulfurospirillum* species compared in this study. This region includes the OHR gene region (for details, see Figure 2). The “Integrated Genome Browser” (v. 9.0.1) was used for data evaluation.

To identify the genes whose transcription is PCE‐dependent, differential RNA sequencing (dRNA‐seq) was performed. The PCE‐respiring *S. multivorans* (DSM 12446^T^) was cultivated with pyruvate as the electron donor and either PCE (Py_PCE) or fumarate (Py_Fu) as the electron acceptor, and per condition, two biological replicates were analyzed. One cDNA library was generated from untreated total RNA (‐TEX), whereas the second library was generated after treatment with terminator exonuclease (+TEX). TEX specifically degrades processed RNAs bearing a 5'‐monophosphate (Sharma et al., 2010; Sharma & Vogel, 2014). The sequencing of these libraries leads to a characteristic enrichment of cDNA reads at the transcriptional start sites (TSSs) in the TEX‐treated sample, which allows for an exact determination of TSSs in a given genome. The cDNA libraries (‐TEX and +TEX) obtained from the two biological replicates (A and B) for each growth condition were sequenced and showed a highly similar transcription pattern (Figure 2; Figure A3). The total number of reads in all eight samples averaged to an amount of six million per library (Table A7). In the presence of PCE, the OHR gene region was transcribed, while in cells cultivated for more than 100 generations with fumarate instead of PCE, most of the transcription stopped in this small section of the genome. The genes with the most notable changes in the transcript level were located in the OHR gene region (see next paragraph). Only two genes outside the OHR gene region displayed a positive log2 fold change (lfc) of at least 3.32 (corresponding to more than 10‐fold higher transcript abundance in Py_PCE compared with Py_Fu): the gene encoding the heat‐shock protein Hsp20 (SMUL_0547) and the gene encoding the periplasmic‐binding protein of a metal transporter (SMUL_0188; Table A8). The three genes with the highest decrease in the transcript level form a single transcriptional unit and encode for a dicarboxylate transporter (SMUL_2818, lfc −8.75), asparaginase (SMUL_2819, lfc −8.92), and an aspartate ammonia‐lyase (SMUL_2817, lfc −9.11), respectively. Also, genes encoding proteins involved in fumarate utilization were substantially less abundant in the presence of PCE, the fumarate hydratase genes (SMUL_1679 and 1680; lfc −3.78 and −3.63, respectively) and an adjacent dicarboxylate transporter gene (SMUL_1681, lfc −4.00).

### 3.3 Transcription of the OHR gene region in *S. multivorans*

**FIGURE A4:**
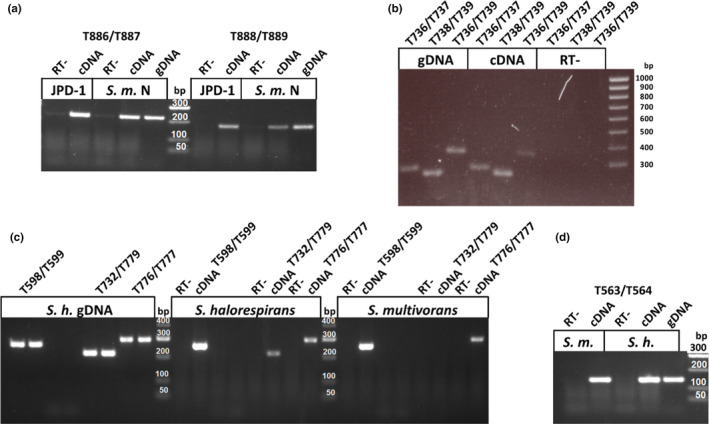
Agarose gels of amplified cDNA. All separations were performed on 2% agarose gels after RT‐PCR. cDNA: sample cDNA; gDNA: genomic DNA positive control; H_2_O: no template negative control; RT‐: no reverse transcriptase negative control; Txyz: primer ID (cf. Table 1). (a) RT‐PCR of RR2 sequences up‐ (T886/T887) and downstream (T888/T889) of the transposases in *S*. sp. JPD‐1 and *S*. *multivorans* strain N. The PCR was stopped after 26 cycles. (b) RT‐PCR of the 106‐nt sequence using different primers in *S*. *halorespirans*. The PCR was stopped after 21 cycles. (c) RT‐PCR of two sequences located at the end of the seventh transcript in the OHR gene region that includes *pceMN* and the genes for the norcobamide biosynthesis. The fragment obtained by using primers T776/T777 covers part of the gene encoding the precorrin‐2 dehydrogenase and *tetR* in *S*. *multivorans* (SMUL_1568 and SMUL_1569) or *S*. *halorespirans* (SHALO_1532 and SHALO_1533). The second fragment (T732/T779) covers part of the Fe‐S cluster binding motif containing the flavoprotein gene (SMUL_1573 or SHALO_1534) and its promoter region. The PCR was stopped after 21 cycles. The 16S rRNA (T598/T599) served as positive control, and the PCR was stopped after 16 cycles. (D) RT‐PCR of the Fe‐S cluster binding motif‐containing flavoprotein in *S*. *multivorans* (SMUL_1573) and *S*. *halorespirans* (SHALO_1543). PCR was stopped after 21 cycles.

Most of the transcripts in the OHR gene region are significantly more abundant in the presence of PCE with an lfc >3.32 (37 out of 44 genes, Figure 2a; Table A9, link to the data set: https://doi.org/10.5281/zenodo.4059358). Exceptions are *rdhB* and the transcripts of the TCS1 histidine kinase and RR2, which are also more abundant in Py_PCE, but not significantly (lfc 0.34/1.65/0.89, Benjamini–Hochberg‐corrected *p*‐values (*p*adj) 0.96/0.15/0.093, respectively). However, the histidine kinase of TCS2, which shares a single transcript with RR2, is significantly more abundant (lfc 1.69, *p*adj 1.5 ∙ 10^−5^; Figure 2b; Table A9, link to the data set: https://doi.org/10.5281/zenodo.4059358). In the absence of PCE, only TCS2 genes are transcribed in the OHR gene region (Figure 2b; Table A9, link to the data set: https://doi.org/10.5281/zenodo.4059358). The TSS of TCS2 is identical in cells cultivated with or without PCE indicating the absence of an alternative promoter for basal expression. The basal transcription was also detected in the non‐dechlorinating *S. multivorans* strain N and *S*. sp. JPD‐1 using RT‐PCR (Figure A4a). In contrast to TCS2, the transcript abundance of TCS1 in *S. multivorans* was low under both conditions Py_PCE and Py_Fu (Figure 2b; Table A9, link to the data set: https://doi.org/10.5281/zenodo.4059358).

The dRNA‐seq results allowed for the determination of eight TSSs in the OHR gene region of *S. multivorans* (Table A10). The first transcript covered a single gene encoding an alkylhydroperoxidase AhpD family protein (SMUL_1530), whose transcriptional level was comparably low. The second unit comprised *pceAB*. The third transcript encoded an IscU/NifU‐like protein (SMUL_1533), followed by the transcripts of TCS1 and TCS2. As mentioned earlier, the *rdhAB* gene cluster showed no transcription. The sixth small transcript covered the gene encoding a putative membrane protein (SMUL_1540, 111 amino acids), which was not detected in any of the proteomes so far. It is predicted to contain three transmembrane helices and is related to bacterial cytochrome *b* of NiFe hydrogenases. It belongs to the DUF4405 (pfam14358) protein family, which contains two conserved histidine residues. The conserved histidine residues at positions 37 and 40 are predicted to face the periplasm close to or at the beginning of the second transmembrane helix. Similar proteins (35% to 50% amino acid sequence identity) are found in different genomic contexts mainly in Proteobacteria and the flavobacterial genus *Lutibacter*. The seventh, very long transcriptional unit (29 genes, approximately 25 kb) contains genes encoding components of a putative quinol dehydrogenase protein complex (*pceMN*; Kruse et al., 2016), as well as genes for the *de novo* norcobamide biosynthesis. The location of both gene regions on a single large transcript was verified by RT‐PCR for *S. halorespirans*, too (Figure A4b). The intact *tetR* gene of *S. halorespirans* is also part of this long transcript (Figure A4c). In *S. halorespirans*, the transcription of the seventh transcriptional unit also spans the intergenic region between *tetR* and the eighth transcriptional unit as quantified by RT‐qPCR (Table A11). However, the transcript level is substantially decreasing (more than two orders of magnitude) in this section, which strongly supports the assumption that this long transcript ends here. The transposases located within the *tetR* gene of *S. multivorans* were not transcribed, but we could detect a putative antisense RNA with an unknown function. The eighth transcript in the OHR gene region of *S. multivorans* included four genes encoding a putative iron–sulfur cluster‐containing flavoprotein (SMUL_1573), a Rieske‐like putative redox protein (SMUL_1574), a putative FMN‐binding protein (SMUL_1575), and a putative membrane protein (SMUL_1576). This region was also transcribed in *S. halorespirans* (Figure A4d).

**FIGURE A5:**
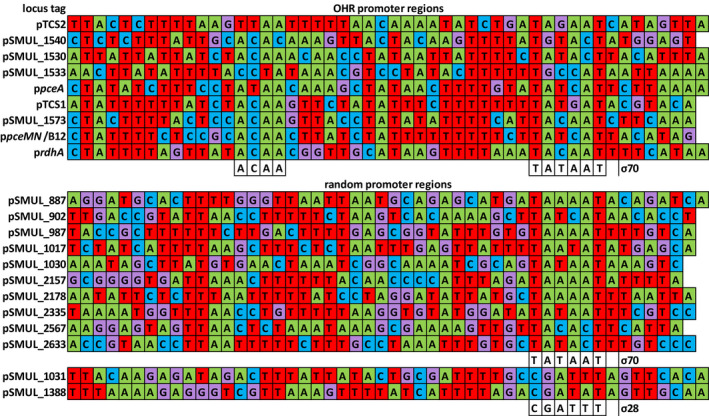
Alignment of the OHR promoter regions and randomly chosen promoter sequences in *S*. *multivorans*. The sequences are up to 50 bp long and the 3'‐end is the defined TSS (+1). The promoter region of *rdhAB* was predicted from sequence alignments.

**FIGURE A6:**
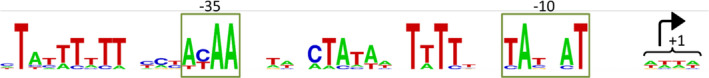
WebLogo of the promoter regions of the eight PCE‐dependent regulated transcripts. The −35 and −10 boxes are highlighted. The TSSs vary between four bp as it is shown in Figure A5.

The promoter sequences of the OHR gene region differed from promoter sequences in the remaining *S. multivorans* genome (Figure A5). The −35 box was identified as ACAA (Figure A6). The −10 box sequence TANNAT displayed similarities to the sequence bound by the sigma factor RpoD of *Escherichia coli* (TATAAT; Pribnow, 1975; Siebenlist et al., 1980). RpoD is the primary sigma factor during exponential growth (Gruber & Gross, 2003), which is in accordance with OHR gene expression in this growth phase. The alignment of 10 randomly chosen RpoD‐dependent promoters in the genome showed a conserved −10 box, whereas the −35 box of this group of genes seemed to be less conserved and more similar to the consensus sequence of *Campylobacter jejuni* (TTTAAGTNTT; Wösten et al., 1998). The consensus sequence of the −35 box of the OHR gene region appeared to be unique for this set of transcriptional units. To further characterize this set of potentially coregulated gene clusters, binding studies of the regulator protein RR2 and various promoter DNA sequences have been conducted.

### 3.4 Electrophoretic mobility shift assays with purified RR2

The molecular details of the OHR gene regulation and the role of TCS2 were further investigated in *S. halorespirans*, because transposase genes are absent in its OHR gene region and the 106 nucleotides upstream of *cbiB* are present (Figure 1). The locus tags for the OHR genes in *S. halorespirans* are given in Table A5. The promoter sequences within the OHR gene region were almost 100% conserved among the dehalogenating *Sulfurospirillum* spp. Because of the high conservation of the OHR gene region's regulatory elements among *Sulfurospirillum* spp., the results obtained for *S. halorespirans* are most likely transferable to the other species. Only the −10 box of the last transcriptional unit, which encodes the flavoproteins and proteins of unknown function, differs in both "*Candidatus* S. diekertiae" strains. Instead of TACAAT in *S. multivorans* and *S. halorespirans*, TAAAAT was identified.

**FIGURE A7:**
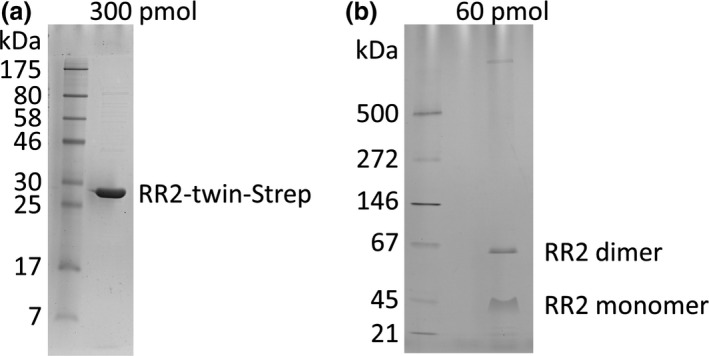
Gel electrophoresis of the purified RR2‐twin‐Strep. (a) Coomassie‐stained denaturing and (b) silver‐stained native polyacrylamide gels

The RR2 protein of *S. halorespirans* was heterologously produced in *E. coli* and purified via affinity chromatography (Figure A7a). In most RR transcription factors, phosphorylation mediates the dimerization of the receiver domains, which is thought to promote DNA binding and transcription activation (Gao & Stock, 2010). Although RR2 might require phosphorylation *in vivo*, the purified protein forms such active dimeric conformations possibly due to the high concentration of the RR2 sample (>25 pmol/µl). The sample contained the inactive monomeric and the dimeric form of the protein, which should be capable of binding DNA (Figure A7b). Since dimer formation already occurred in a sufficient amount, an additional phosphorylation step was not required *in vitro*. The binding of the regulator to the OHR promoter sites was analyzed in electrophoretic mobility shift assays (EMSAs) using fluorescently labeled DNA fragments containing the various promoter sequences. To ensure the integrity of every promoter sequence, the DNA fragments covered a region from 150 bp upstream of the TSS to the start codon of the first gene of the transcriptional unit. The promotor of TCS1 and the promotor of *rdhAB* are located in the same intergenic region because the neighboring gene clusters are orientated differently. This configuration was also found for the promotor sites of TCS2 and the neighboring SHALO_1504.

**FIGURE 3:**
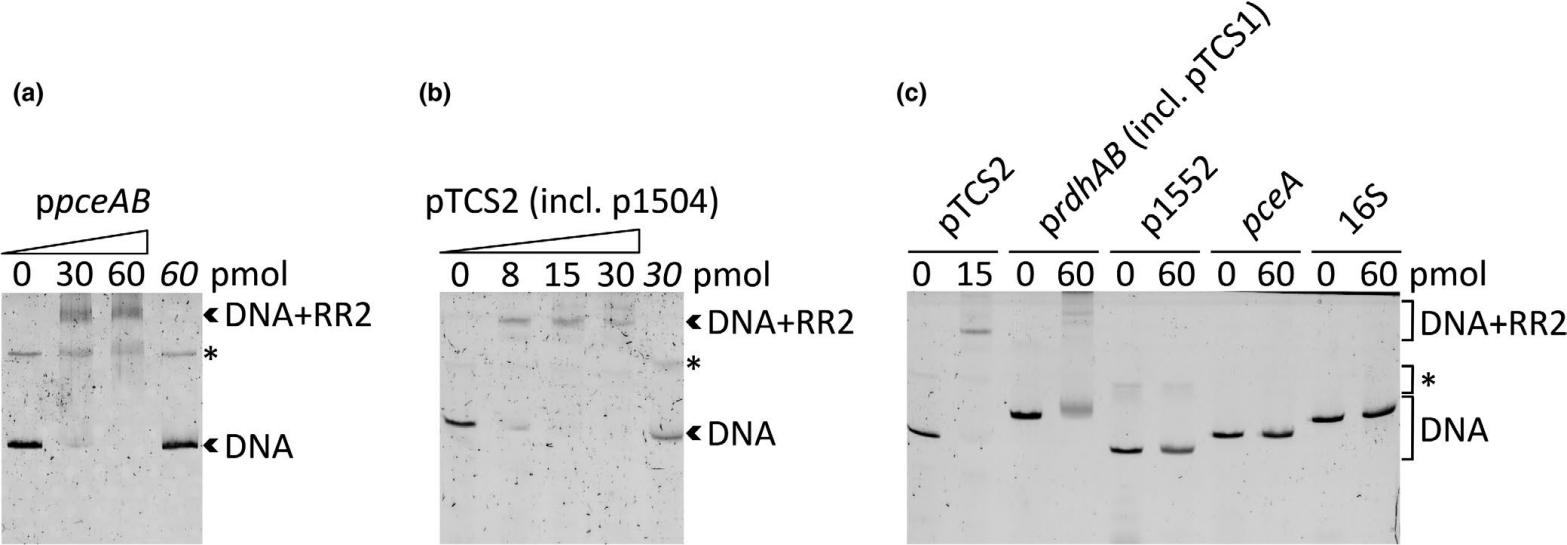
Gel shift assays of *Sulfurospirillum halorespirans* RR2‐twin‐Strep binding to the promoters of (a) p*pceAB* and (b) the intergenic region between TCS2 and SHALO_1504. As a negative control (last lane in both cases with the amount of protein given in italics), the sample mixture was boiled for 5 min after the completed binding reaction. (c) Control reactions for the gel shift assay with pTCS2. The promoters p*rdhAB* and pTCS1 are combined in a single intergenic region. Promoter p1552 is located in the flanking region downstream of the OHR gene region and belongs to a gene encoding for a phage CI repressor superfamily protein. Short DNA stretches from the coding sequences of the *pceA* and the 16S rRNA genes were used. The amount of fluorescently labeled DNA was 2.5 pmol, whereas the protein amount was increased in each lane as indicated. A DNA artifact not targeted by RR2 is marked by an asterisk.

**FIGURE A8:**
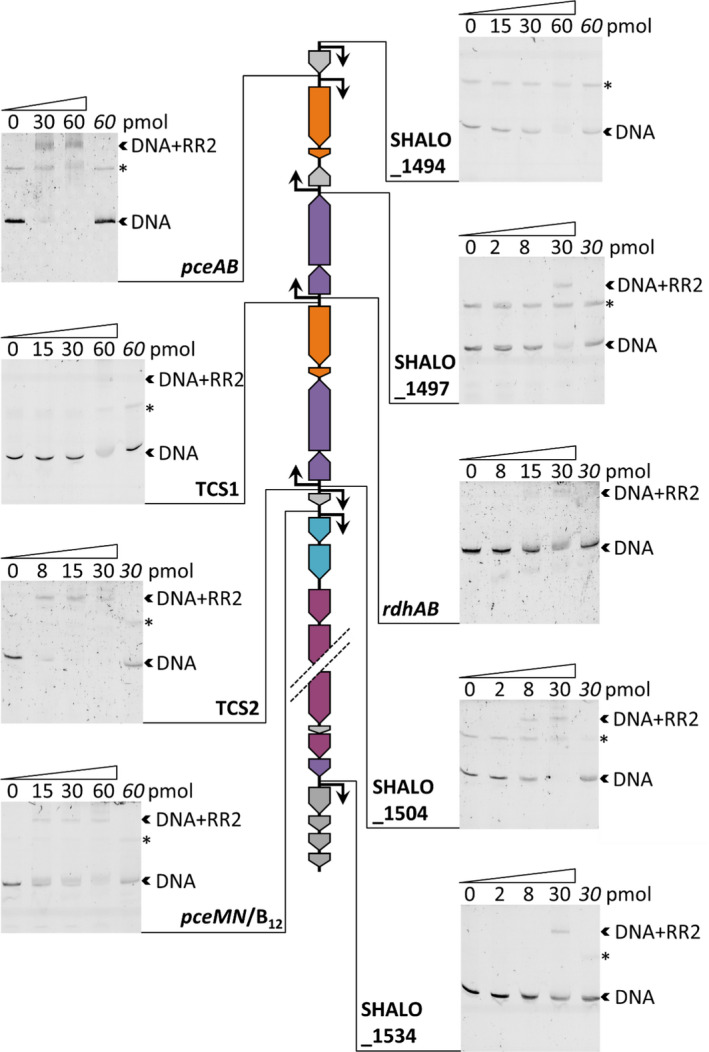
Gel shift assays of *S*. *halorespirans* RR2‐twin‐Strep binding to the promoters of all transcriptional units of the OHR gene region. The amount of fluorescently labeled promoter DNA was 2.5 pmol, whereas the protein amount increased in each lane as indicated. As a negative control (protein amount written in italics), the sample mixture was boiled for 5 min after the binding reaction. A DNA artifact not targeted by RR2 is marked by an asterisk.

Using 2.5 pmol fluorescently labeled DNA, shifts were detected in the cases of *pceAB* and TCS2 (incl. SHALO_1504) after adding different amounts of purified RR2 (Figure 3a,b). The addition of triethylene glycol was necessary to stabilize protein–DNA complexes and to reduce their dissociation during electrophoresis. A second DNA band appeared upon the addition of triethylene glycol, but the intensity of this band did not change when RR2 was added. In general, between 8 and 60 pmol of RR2 were required to visualize complex formation with promoter regions of the OHR gene region (Figure A8). For selected promoter regions (SHALO_1494, *rdhAB*, and TCS1), the protein–DNA complex band was barely visible, although the DNA band disappeared at higher protein concentrations. After boiling, the protein‐free DNA reappeared, which was interpreted as a result of the dissociation of the RR2‐DNA complex. A set of negative controls confirmed the specific binding of RR2 (Figure 3c). DNA sequences amplified from the *pceA* structural gene, the 16S rRNA gene, or the promoter sequence of a gene encoding for a phage CI repressor superfamily protein (SHALO_1552, located downstream of the OHR gene region) did not interact with RR2. All assays performed indicate that the regulator binds to all promoter sites of the OHR gene region that have been predicted by RNA sequencing before.

**FIGURE 4:**
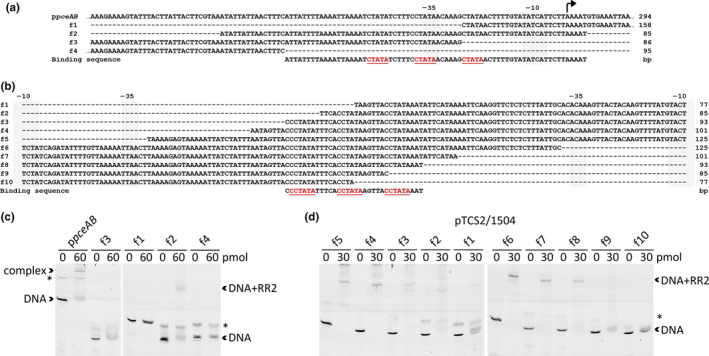
*4*Truncated promoter DNA sequences (f1–10) of (a) p*pceAB* and (b) pTCS2/1504 are shown together with the respective gel shift assays performed with RR2 and the DNA fragments of (c) p*pceAB* and (d) pTCS2/1504. The direct repeat in the suggested binding sequence is underlined. The amount of 6Fam‐labeled promoter DNA was 2.5 pmol, whereas the protein amount was 0, 30, or 60 pmol as indicated. A DNA artifact not targeted by RR2 is marked by an asterisk. See also Figure A10 for further EMSA analyses of the promoter sequences of *pceMN*/B_12_, SHALO_1534, SHALO_1494, SHALO_1497, and TCS1.

**FIGURE A9:**
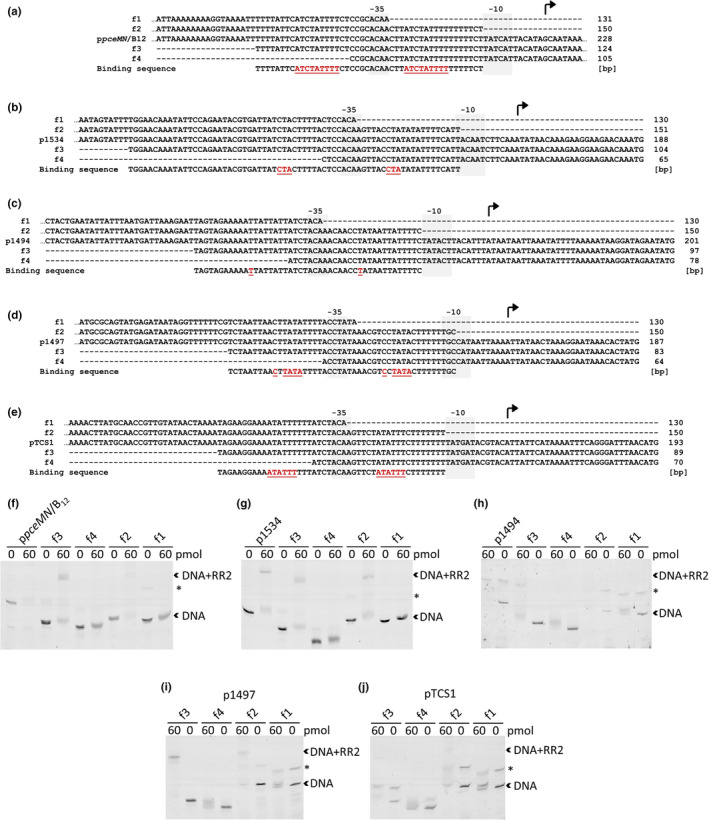
Truncated promoter DNA sequences (f1‐4) of (a) p*pceMN*/B_12_, (b) p1534, (c) p1494, (d) p1497, and (e) pTCS1 are shown together with the respective gel shift assays performed with RR2 and the DNA fragments of (f) p*pceMN*/B_12_, (g) p1534, (h) p1494, (i) p1497, and (j) pTCS1. The direct repeat in the suggested binding sequence is underlined. The amount of 6Fam‐labeled promoter DNA was 2.5 pmol, whereas the protein amount to 0 or 60 pmol, as indicated. A DNA artifact not targeted by RR2 is marked by an asterisk. See also Figure 5 for further EMSA analyses of the promoter sequences of p*pceAB* and pTCS2/1504.

RR2 has a predicted OmpR‐family domain structure. Thus, it was assumed that an RR2 dimer pair binds a direct repeat. To identify and localize the cis‐regulatory elements (CREs) bound by RR2, a screening of the promotor sequences with short overlapping DNA fragments was conducted. In the case of the intergenic region upstream of *pceAB*, which showed the highest upregulation in the dRNA‐seq, the minimum binding sequence defined by EMSA was a 70‐bp DNA fragment (Figure 4a,c). This DNA fragment harbors short direct repeats overlapping the −35 box. A sequence of CTATA is repeated twice with a gap of six nucleotides, respectively. While the central CTATA is located in the −35 box and is probably bound by the σ^70^ factor, the first and last CTATA are available for binding an RR2 dimer. Except for the promoters of TCS2 and SHALO_1504, all the CREs overlap the −35 box of the promoters. These promoters lost the capacity to be bound by RR2 when either repeat, up‐ or downstream of the −35 box, was absent (Figure A9)—a result that disfavors the binding of monomeric RR2.

The intergenic region between TCS2 and SHALO_1504 does not have two separate CREs, but a single CRE located 85 bp upstream of both TSSs in the center of this intergenic region (Figure 4b,d). This region also contains three repeats of the CTATA motif with an additional C at the 5′ end, respectively. In contrast to the promoters analyzed before, the central repeat will not be bound by the RNA polymerase and is therefore available for binding of RR2. When the intergenic region between TCS2 and SHALO_1504 was tested, the gel shift was larger compared with the other DNA‐RR2 complexes indicating that probably more than a single RR2 dimer binds this CRE and promotes transcriptional activation of both operons. This assumption was supported by the fact that the shift was remarkably lower when the first CCTATA sequence at the CRE’s 5′ end was incomplete (Figure 4d, f2). The repeat closest to the SHALO_1504 TSS is essential for RR binding at all (Figure 4d, f9).

**FIGURE 5:**
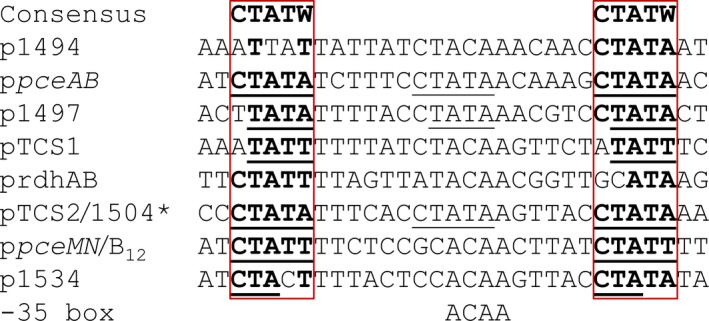
Sequence alignment of the CREs in the OHR gene region. The promoter DNA sequences of all transcriptional units and the putative promoter sequence of the *rdhAB* genes are listed. The CRE, which is located 85 bp upstream of the TSS, is labeled with an asterisk. The −35 box with the consensus sequence for the binding of the σ^70^ factor (RpoD) is given below (gray background). The suggested consensus sequence of the CREs was determined to be CTATW‐N_17_‐CTATW. Nucleotides that fit the consensus sequence are printed in bold. Direct repeats (≥3 nucleotides) that match the consensus sequences are underlined.

Sequence alignment of the CRE of these two promoters with the six CREs of the promoters overlapping the −35 box revealed a CTATW consensus sequence, which is separated by a gap of 17 bp (Figure 5). The promoter sequences of the genes with a comparably low transcript level showed a higher divergence in this repeat. The promoters of the *ahpD*‐like gene, p1494, as well as p*rdhAB*, showed the highest discrepancy, which might be the reason for the weak interaction with RR2 in EMSA in both cases. It might further explain the weak upregulation of SHALO_1494 in dehalogenating *Sulfurospirillum* spp. cultivated with PCE and the absence of RdhA in such cells. Taken together these findings, the data strongly support the assumption of a PCE‐dependent regulon for OHR in *Sulfurospirillum* spp.

## 4 DISCUSSION

The expression of the OHR gene region in *Sulfurospirillum multivorans* is inducible by PCE or TCE (John et al., 2009). However, the cellular components responsible for PCE‐sensing and regulation of OHR were not known. In the genomes of *S. multivorans* strain N and *S*. sp. JPD‐1, which are unable to dechlorinate PCE, the response regulator gene of TCS2 was disrupted by transposase genes. While *S. multivorans* strain N was isolated as a non‐dechlorinating strain, *S*. sp. JPD‐1 was initially characterized as PCE‐dechlorinating (Pietari, 2003) but might have lost this physiological trait during strain conservation. The insertion of transposase genes into RR2 might be a general evolutionary mechanism to shut down PCE‐dependent regulation in *Sulfurospirillum* spp., albeit more long‐term population studies are necessary to confirm this hypothesis. The high conservation of the whole OHR gene region is unusual, but it is still difficult to postulate the underlying molecular mechanisms and to shed light on the reason for this invariability. While a (possibly horizontal) gene transfer of parts of the OHR region from an unknown donor is possible (Goris et al., 2014), very recent horizontal gene transfer (i.e., during the last decades) is unlikely given the geographical distance of the species’ habitats. This accounts especially for *S*. sp. JPD‐1, which was isolated in North America, while the other *Sulfurospirillum* spp. were isolated in Europe. Also, the variability of *pceA* gene sequences (and its surrounding regulatory elements) among *Sulfurospirillum* spp. argue against the possibility of a very recent gene transfer. Opposed to the situation in *D. mccartyi* (Kube et al., 2005; McMurdie et al., 2011; Molenda et al., 2019; Türkowsky, et al., 2018), there are no indicators of the *rdh* genes located on mobile genomic islands or similar elements pointing to a recent horizontal gene transfer (Goris, et al., 2015). This evolutionary hot spot in the *pceA* gene and its manifestation of amino acid changes in PceA might reflect an adaptation of the bacterial lifestyle and the enzyme's substrate range to a certain ecophysiological role as observed in the two populations of "*Candidatus* S. diekertiae" (Buttet et al., 2018). As reported, small changes in the amino acid composition of reductive dehalogenases may lead to a change in the substrate preferences (Kunze et al., 2017b; Schubert et al., 2018).

Using dRNA‐seq, eight transcriptional units were identified in the transcriptome of the PCE‐respiring *S. multivorans* that originated from the OHR gene region. The different transcript levels of those transcripts were in accordance with the protein levels detected in proteome analyses of *S. multivorans* (Goris, et al., 2015) and *S. halorespirans* (Türkowsky, et al., 2018) and with the PCE reductive dehalogenase among the most abundant transcripts and proteins. Only a few transcripts outside the OHR gene region showed substantial differences in the abundance under Py_PCE versus Py_Fu growth conditions, which was also in agreement with previous proteomic studies (Goris, et al., 2015; Türkowsky, et al., 2018). One example is Hsp20, which was discussed as a stress response protein before. While the detection of small membrane‐integral proteins remains difficult in proteomic studies, the transcription of the corresponding genes was detectable in the RNA‐seq data. For example, the expression of the small putative membrane protein encoded by SMUL_1540 with an unknown function was observed for the first time.

In the absence of PCE, the transcription of the OHR genes stopped, while the TCS2 operon was still transcribed at a low level. This observation was in accordance with the detection of the TCS2 gene products in *S. multivorans* and *S. halorespirans* cells cultivated in the absence of PCE (Goris, et al., 2015; Türkowsky, et al., 2018). These data further supported the assumption that TCS2 is crucial for the transcriptional response to PCE in *Sulfurospirillum* spp. There is no indication that the TetR‐like regulator in *S. halorespirans* affects gene regulation of the OHR gene region. The TCS2 genes are not located directly adjacent to the reductive dehalogenase gene, whose expression is regulated by TCS2. Such a situation is uncommon in organohalide‐respiring bacteria studied so far (Maillard & Willemin, 2019). Other organohalide‐respiring bacteria harbor different signaling systems that sense environmental halogenated organic compounds (Gábor et al., 2008; Kruse et al., 2017; Maillard & Willemin, 2019; Türkowsky, et al., 2018), with TCS genes encoding putative cytoplasmic sensor variants present only in Dehalococcoidia (Wagner et al., 2013). Whether the presence of organohalides is directly sensed by Dehalococcoidia or indirectly monitored via other signals such as the redox state of the cell is unknown (Krasper et al., 2016; Kube et al., 2005; Wagner et al., 2013). OHR regulation via membrane‐associated TCSs is found in *Sulfurospirillum* spp. and probably in a few *Desulfitobacterium* species, in which TCS genes are encoded adjacent to unstudied *rdh* genes (Kruse et al., 2017). Outside the genus *Sulfurospirillum*, TCS2 exhibits the highest protein sequence identity (histidine kinase: 35%; RR: 46%) to a TCS with unknown function in *Arcobacter ebronensis* (Campylobacterota).

**FIGURE 6:**
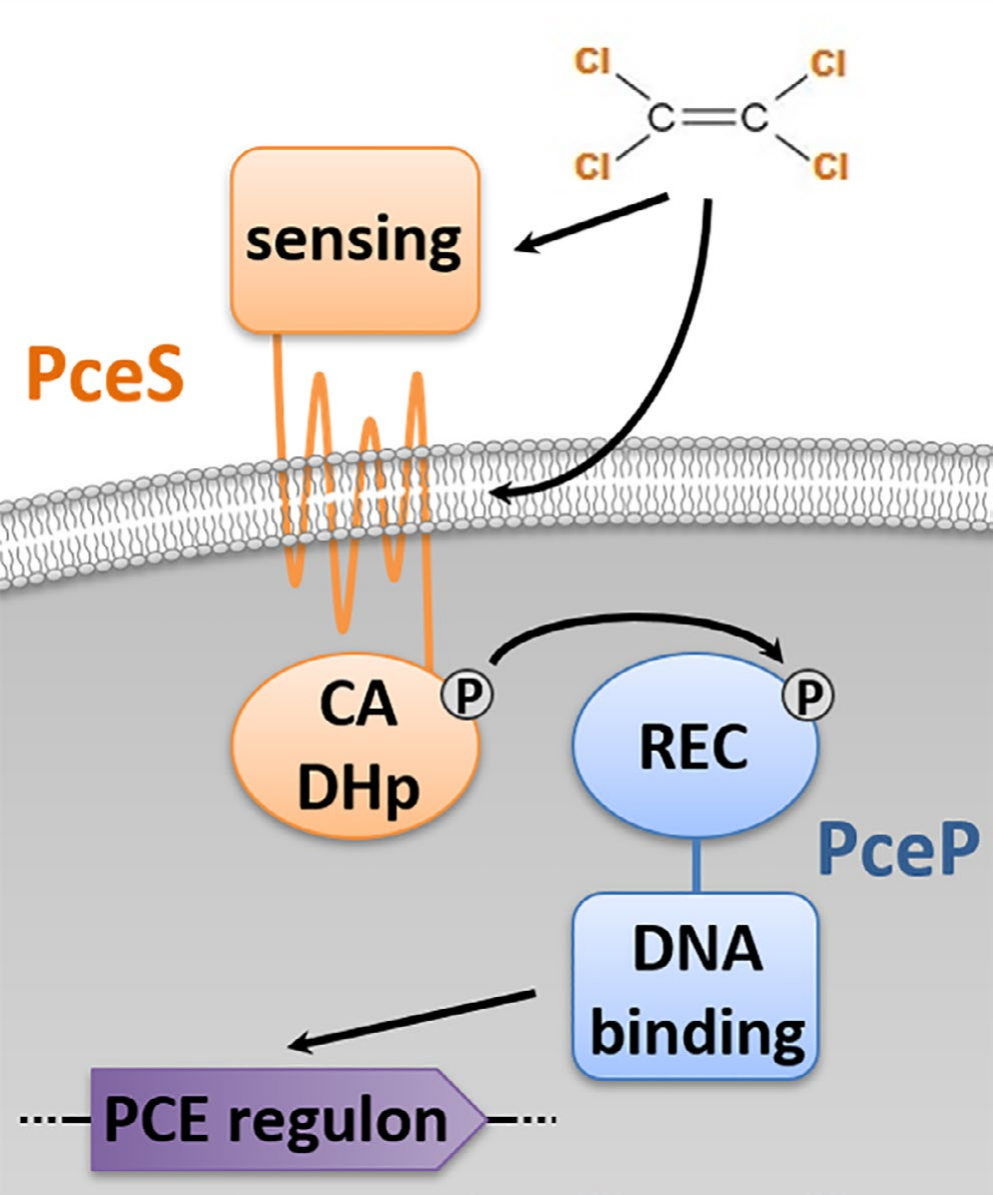
Tentative scheme of the signal transduction by TCS2 in *Sulfurospirillum* spp.

Following the nomenclature of OHR genes (Kruse et al., 2016), RR2 and the cognate histidine protein kinase encoded in the OHR gene region of *Sulfurospirillum* spp. were designated as PceP and PceS, respectively. To derive a first model for PCE‐sensing in the dehalogenating *Sulfurospirillum* spp., the domain structures of PceP and PceS were predicted (Figure 6). PceS contains a putative periplasmic N‐terminal sensing domain and an extensive transmembrane domain with seven transmembrane helices. One of the two domains might be involved in the interaction with the hydrophobic PCE. The cytoplasmic C‐terminus of PceS consists of the dimerization and histidine phosphotransfer domain (DHp) and the catalytic and ATP‐binding domain (CA). PceP belongs to the OmpR‐family of regulator proteins and contains a receiver (REC) domain and a DNA‐binding domain with a winged helix–turn–helix (wHTH) motif (Figure 6). The REC domain contains a highly conserved aspartate residue. This residue is proposed to be phosphorylated in the presence of PCE. Although phosphorylation of PceP was not required for dimerization and DNA binding *in vitro*, possibly due to its high concentration in the purified sample, phosphorylation is expected to promote efficient dimerization *in vivo*, when PceP concentrations are comparably low. Since phosphorylation of PceP was not achieved *in vitro*, its impact could not be examined in this survey. Hence, a non‐canonical response to PCE, that is, without phosphotransfer, cannot be ruled out. However, typical non‐canonical TCSs are orphan RRs, RRs lacking the conserved aspartate residue, or TCSs with an excess of HK or an HK primarily serving as a phosphatase (Desai & Kenney, 2017). PceP and PceS do not correspond to these categories and are instead more related to classical TCSs.

The OmpR‐like regulator of TCS2 seems to promote two different transcriptional activation mechanisms, which is unusual for RRs. In the case of pTCS2/p1504, when PceP binds the CRE 85 bp upstream of the two TSSs, it is predicted to promote transcription initiation by recruiting the RNA polymerase. This mechanism is comparable to other OmpR‐family regulators (Martínez‐Hackert & Stock, 1997). A repressing effect of PceP at this location is rather unlikely. In the presence of PCE, PceP functions as an activator for its transcription resulting in an increased amount of *pceP* transcript and a high PceP level. The raised PceP level also favors an activation mechanism for the other promoters although the CREs overlap the −35 box. A repression/derepression mechanism as one might assume first is not feasible. An elevated level of PceP would promote repression in the presence of PCE, but this has not been observed. In addition, it is very unlikely that PceP acts on the one hand as an activator and on the other hand as a repressor by binding the consensus sequence of different promoters. It is assumed that the PceP activates transcription by interacting with the sigma factor and assisting the isomerization of an initially closed into an open complex of RNA polymerase and promoter DNA. However, further experiments are required to finally resolve the molecular mechanism of PceP‐mediated gene regulation.

In the absence of PCE, the TCS2 gene cluster showed basal transcription. The DNA sequences adjacent to the −35 box of pTCS2, which are not involved in the binding of PceP in this specific case, differ from the sequences of the other promoters in the OHR gene region (Figure A5). The basal transcription of the TCS2 operon might be a result of this alternative promoter architecture. Ongoing studies include the mutagenesis of the promoter sequences of the OHR gene region. This will shed more light on the reason for the PCE‐independent basal transcription of TCS2 and the role of the CRE sequence variations in all PCE‐dependent promoters, which are suggested to balance OHR gene expression in the PCE regulon of dehalogenating *Sulfurospirillum* spp.

## ETHICS STATEMENT

None required.

## CONFLICT OF INTEREST

None declared.

## AUTHOR CONTRIBUTION

**Jens Esken:** Investigation (equal); Methodology (equal); Visualization (equal); Writing‐original draft (equal); Writing‐review & editing (equal). **Tobias Goris:** Conceptualization (equal); Data curation (equal); Formal analysis (equal); Investigation (equal); Methodology (equal); Project administration (equal); Validation (equal); Visualization (equal); Writing‐original draft (equal); Writing‐review & editing (equal). **Jennifer Gadkari:** Investigation (equal). **Thorsten Bischler:** Data curation (equal); Formal analysis (equal); Investigation (equal); Visualization (equal); Writing‐review & editing (equal). **Konrad Förstner:** Formal analysis (equal); Investigation (equal); Visualization (equal); Writing‐review & editing (equal). **Cynthia Sharma:** Methodology (equal); Resources (equal); Supervision (equal); Validation (equal); Writing‐review & editing (equal). **Gabriele Diekert:** Funding acquisition (equal); Resources (equal); Supervision (equal); Writing‐review & editing (equal). **Torsten Schubert:** Conceptualization (equal); Funding acquisition (equal); Project administration (equal); Validation (equal); Visualization (equal); Writing‐review & editing (equal).

## Data Availability

The genomes of *S. multivorans* strain N and *S*. sp. JPD‐1 are available at NCBI under the GenBank accession numbers CP042966: https://www.ncbi.nlm.nih.gov/nuccore/CP042966 and CP023275: https://www.ncbi.nlm.nih.gov/nuccore/CP023275, respectively. RNA‐seq data are available at NCBI Gene Expression Omnibus (GEO; Barrett et al., 2012) under accession number GSE139083: https://www.ncbi.nlm.nih.gov/geo/query/acc.cgi?acc=GSE139083. The table presenting the DeSeq2 results of the *S. multivorans* Py_PCE_minus_Tex replicates (A and B) vs. the Py_Fu_minus_Tex replicates (A and B) is publicly available in the Zenodo repository at https://doi.org/10.5281/zenodo.4059358.

## Appendix 1

### 1.1

**TABLE A1:** List of oligonucleotides used in RT‐PCR.

Oligo	Sequence (5ʹ‐>3ʹ)	Gene	Name
T563	CCGGCCTGCAACACATGAGCCTAA	SMUL_1574	
T564	CCGGAAGGAAGGCTTTTCCCTTCA	SMUL_1574	
T598	GAGACACGGTCCAGACTCCTAC	SMUL_2335/SMUL_3269 SHALO_2082/SHALO_2989	*rrsA1/rrsA2*
T599	CTCGACTTGATTTCCAGCCTAC	SMUL_2335/SMUL_3269 SHALO_2082/SHALO_2989	*rrsA1/rrsA2*
T732	CGAATTAATTATACAAACCATACTA	SMUL_1573	
T736	CATTGAAACAATGAATAAAACAGC	SMUL_1542	
T737	AAGACATTGTATTAATGCAACGTG	SMUL_1542	
T738	CCGTTAGAACAATATTTTTTTCTG	SMUL_1543	*cbiB*
T739	GCAAAAAAAGCTATTAATGCGG	SMUL_1543	*cbiB*
T774	AATATTGCAAAAACATTAGAAGAG	SMUL_1540	
T775	ATTAAAAATTTTCTTCGATTAAAATTC	SMUL_1541	
T776	TTAATGTATTTGTAAAACAGCAC	SMUL_1568	*sirC*
T777	TTCCCAAAAAAGTTCCATTGC	SMUL_1569	
T778	AACCATACTAGCACTGCTC	SMUL_1572	
T779	GGTGAGCCGGATACTCC	SMUL_1573	
T886	TATGCAGAAGATGATGCAGG	SMUL_1539	*pceP*
T887	GTAGCCATGACAATCGGG	SMUL_1539	*pceP*
T888	TATATGTTGAAAAATCCTAATAGAG	SMUL_1539	*pceP*
T889	TGAAGAACCAGCTTGTATCC	SMUL_1539	*pceP*

**TABLE A2:** List of oligonucleotides used for cloning.

Oligo	Sequence (5ʹ‐>3ʹ)	Gene	Name
	Cloning primer pair		
T906	AGCGCGTCTCCAATGTTTAAAAACTATAAAGTACTTTATGCAGAAG	SHALO_1503	*pceP*
T907	AGCGCGTCTCCTCCCTCATTTTTGAAGAACCAGC	SHALO_1503	*pceP*
	DNA sequencing primers		
T954	GAGTTATTTTACCACTCCCT	pASG‐IBA105 (SHALO_1503)	*pceP*
T955	CGCAGTAGCGGTAAACG	pASG‐IBA105 (SHALO_1503)	*pceP*

**TABLE A3:** List of oligonucleotides used for EMSA.

Oligo	Sequence (5ʹ‐>3ʹ)	Gene	Name
T599	CTCGACTTGATTTCCAGCCTAC	SHALO_2082/SHALO_2989	*rrsA1/rrsA2*
T898	6‐FAM‐GAGACACGGTCCAGACTCCTAC	SHALO_2082/SHALO_2989	*rrsA1/rrsA2*
T982	6‐FAM‐GCCTTTAGTTCCAGATAAGCCTATCGAC	SHALO_1495	*pceA*
T607	GGCCCGCCACAATATCCACCAGAT	SHALO_1495	*pceA*
T983	6‐FAM‐TTGTATATTTTATTAATATTAGCTTATAAATG	pSHALO_1552	
T984	CATTAAATTCTTAATTACATCATTATATC	pSHALO_1552	
T890	6‐FAM‐TTAAGAAAAAGTTATAGATGTAGAT	pSHALO_1494	
T719	CATATTCTATCCTTATTTTTAAAAT	pSHALO_1494	
T787	6‐FAM‐ATTGTCTGATATATTGACAAAATCT	pSHALO_1495	p*pceA* (x4)
T721	CATACTTCCTCCTTAAAAAATACC	pSHALO_1495	p*pceA* (x1)
T891	6‐FAM‐CAAAATTTATCATTGATCTTAGCC	pSHALO_1497	
T723	CATAGTGTTTATTCCTTTAGTTATA	pSHALO_1497	
T892	6‐FAM‐CCTTCTTGTAACCTTTAATTAAATG	pSHALO_1499	pTCS1
T725	CATGTTAAATCCCTGAAATTTTATG	pSHALO_1499	pTCS1
T893	6‐FAM‐AGTACATAAAACTTGTAGTAACTTT	pSHALO_1503	pTCS2 (x1‐5)
T727	CATGTGTGAACCTATCCTATAG	pSHALO_1503	pTCS2
T894	6‐FAM‐TCTATCAGATATTTTGTTAAAAATT	pSHALO_1504	(x6‐10)
T729	CATGTCGTTCCTTTATTTTTG	pSHALO_1504	
T895	6‐FAM‐CACATAATGAAGGACTTTATGATAA	pSHALO_1505	p*pceMN*/B12 (x1‐2)
T731	CATCTTTTTCTCTCTTAAAGATTGT	pSHALO_1505	p*pceMN*/B12
T896	6‐FAM‐CGAATTAATTATACAAACCATACTA	pSHALO_1534	
T733	CATTTGTTCTTCCTTCTTTGTTATA	pSHALO_1534	(x1‐2)
T897	6‐FAM‐CTGCATAGAGTATTGTATAAGAAG	pSHALO_1500	p*rdhAB*
T735	CATATTTACTCCTTAAAAAATCATC	pSHALO_1500	p*rdhAB*
T986	TAAAAGAGTAAAAATTATCTATTTAATAG	pSHALO_1503/1504	pTCS2/SHALO_1504x5
T987	AATAGTTACCCTATATTTCACCT	pSHALO_1503/1504	pTCS2/SHALO_1504x4
T991	CCCTATATTTCACCTATAAGTTA	pSHALO_1503/1504	pTCS2/SHALO_1504x3
T992	TTCACCTATAAGTTACCTATAAATA	pSHALO_1503/1504	pTCS2/SHALO_1504x2
T993	TAAGTTACCTATAAATATTCATAAA	pSHALO_1503/1504	pTCS2/SHALO_1504x1
T989	GCAATAAAGAGAGAACCTTG	pSHALO_1503/1504	pTCS2/SHALO_1504x6
T990	TTATGAATATTTATAGGTAACTTAT	pSHALO_1503/1504	pTCS2/SHALO_1504x7
T994	ATTTATAGGTAACTTATAGGTG	pSHALO_1503/1504	pTCS2/SHALO_1504x8
T995	GTAACTTATAGGTGAAATATAG	pSHALO_1503/1504	pTCS2/SHALO_1504x9
T996	TAGGTGAAATATAGGGTAACT	pSHALO_1503/1504	pTCS2/SHALO_1504x10
T1006	6‐FAM‐CTATAACTTTTGTATATCATTCTTAAAAT	pSHALO_1495	p*pceAB*x1
T1005	ATATTATTAACTTTCATTATTTTAAAATTAAA	pSHALO_1495	p*pceAB*x2
T1007	6‐FAM‐ATTTTAAGAATGATATACAAAAGTTATAG	pSHALO_1495	p*pceAB*x2
T1002	AAAGAAAAGTATTTACTTATTACTTC	pSHALO_1495	p*pceAB*x3
T997	6‐FAM‐CTTTGTTATAGGAAAGATATAGATT	pSHALO_1495	p*pceAB*x3
T998	GAAAGTTAATAATATTTACGAAGTA	pSHALO_1495	p*pceAB*x4
T1009	TTTTATTCATCTATTTTCTCCGCA	pSHALO_1505	p*pceMN*/B12x3
T1010	CCGCACAACTTATCTATTTTTTTTT	pSHALO_1505	p*pceMN*/B12x4
T1008	6‐FAM‐CATCTTTTTCTCTCTTAAAGATTGT	pSHALO_1505	p*pceMN*/B12 (x3‐4)
T1011	AGAAAAAAAAATAGATAAGTTGTGCG	pSHALO_1505	p*pceMN*/B12x2
T1012	TTGTGCGGAGAAAATAGATGAATA	pSHALO_1505	p*pceMN*/B12x1
T1014	TGGAACAAATATTCCAGAATACGT	pSHALO_1534	pSHALO_1534x3
T1015	CTCCACAAGTTACCTATATATTTT	pSHALO_1534	pSHALO_1534x4
T1013	6‐FAM‐CATTTGTTCTTCCTTCTTTGTTATA	pSHALO_1534	(x3‐4)
T1016	AATGAAAATATATAGGTAACTTGTGG	pSHALO_1534	pSHALO_1534x2
T1017	TGTGGAGTAAAAGTAGATAATCAC	pSHALO_1534	pSHALO_1534x1

**TABLE A4:** Comparison of the five different *Sulfurospirillum* genomes carrying an OHR gene region.

	*S. multivorans*	*S. multivorans* strain N	*S. halorespirans*	*S*. sp. JPD‐1	*“Candidatus* S. diekertiae” SL2‐1*
Strain collection no.	DSM 12446^T^	DSM 15119	DSM 13726	DSM 16452	(11)
GenBank acc. no.	CP007201.1	CP042966	CP017111.1	CP023275	CP021416.1
Genome size (Mbp)	3.18	3.18	3.03	2.81	2.88
GC content (%)	41	41	41	39	39
CRISPR regions	1	1	1	‐	1
rRNA operons	2	3	2	3	3
tRNAs	45	43	41	33	33

Note

The comparison is based on the automated NCBI RefSeq annotation. (*):“*Candidatus* S. diekertiae” includes two strains with a very similar genome, here the complete SL2‐1 genome was chosen as representative. The “*Candidatus* S. diekertiae” strains SL2‐1 (dechlorinating PCE to TCE) and SL2‐2 (dechlorinating PCE to *c*DCE) were enriched from a Dutch bioreactor loaded with PCE‐contaminated water.

**TABLE A5:** Locus tags of the OHR gene region. Functions: gray = unknown; orange = reductive dehalogenase; violet = regulatory proteins; turquoise = putative quinol dehydrogenase; purple = norcobamide biosynthesis; pink = cobamide transporter.

Locus tags				Product
SHALO_1494	SMUL_1530	SMN_1515	SJPD1_1503	alkylhydroperoxidase AhpD family protein
SHALO_1495	SMUL_1531	SMN_1516	SJPD1_1504	tetrachloroethene reductive dehalogenase catalytic subunit PceA
SHALO_1496	SMUL_1532	SMN_1517	SJPD1_1505	tetrachloroethene reductive dehalogenase membrane anchor PceB
SHALO_1497	SMUL_1533	SMN_1518	SJPD1_1506	IscU/NifU‐like protein
SHALO_1498	SMUL_1534	SMN_1519	SJPD1_1507	two‐component sensor histidine kinase
SHALO_1499	SMUL_1535	SMN_1520	SJPD1_1508	two‐component response regulator
SHALO_1500	SMUL_1536	SMN_1521	SJPD1_1509	reductive dehalogenase catalytic subunit RdhA
SHALO_1501	SMUL_1537	SMN_1522	SJPD1_1510	reductive dehalogenase membrane anchor RdhB
SHALO_1502	SMUL_1538	SMN_1523	SJPD1_1511	two‐component sensor histidine kinase
SHALO_1503	SMUL_1539			two‐component response regulator
		SMN_1524	SJPD1_1512	two‐component response regulator, disrupted by transposase
		SMN_1525		transposase
			SJPD1_1513	putative transposase
			SJPD1_1514	putative transposase
			SJPD1_1515	two‐component response regulator, disrupted by transposase
SHALO_1504	SMUL_1540	SMN_1526	SJPD1_1516	putative membrane protein
SHALO_1505	SMUL_1541	SMN_1527	SJPD1_1517	putative quinol dehydrogenase, periplasmic subunit
SHALO_1506	SMUL_1542	SMN_1528	SJPD1_1518	putative quinol dehydrogenase, membrane subunit
SHALO_1507	SMUL_1543	SMN_1529	SJPD1_1519	adenosylcobinamide‐phosphate synthase CbiB
SHALO_1508	SMUL_1544	SMN_1530	SJPD1_1520	threonine phosphate decarboxylase‐like enzyme
SHALO_1509	SMUL_1545	SMN_1531	SJPD1_1521	bifunctional cobamide biosynthesis protein CobU
SHALO_1510	SMUL_1546	SMN_1532	SJPD1_1522	cobyric acid synthase CbiP
SHALO_1511	SMUL_1547	SMN_1533	SJPD1_1523	nicotinate‐nucleotide‐‐dimethylbenzimidazole phosphoribosyltransferase CobT
SHALO_1512	SMUL_1548	SMN_1534	SJPD1_1524	cysteine‐rich domain protein
SHALO_1513	SMUL_1549	SMN_1535	SJPD1_1525	cobamide synthase CobS
SHALO_1514	SMUL_1550	SMN_1536	SJPD1_1526	alpha‐ribazole phosphatase CobC
SHALO_1515	SMUL_1551	SMN_1537	SJPD1_1527	sirohydrochlorin cobaltochelatase CbiK
SHALO_1516	SMUL_1552	SMN_1538	SJPD1_1528	corrinoid ABC transporter, permease component BtuC
SHALO_1517	SMUL_1553	SMN_1539	SJPD1_1529	corrinoid ABC transporter ATPase BtuD
SHALO_1518	SMUL_1554	SMN_1540	SJPD1_1530	corrinoid ABC transporter, B12‐binding component BtuF
SHALO_1519	SMUL_1555	SMN_1541	SJPD1_1531	cobalt‐precorrin‐8x methylmutase CbiC
SHALO_1520	SMUL_1556	SMN_1542	SJPD1_1532	cobalt‐precorrin‐6 synthase, anaerobic CbiD
SHALO_1521	SMUL_1557	SMN_1543	SJPD1_1533	cobalt‐precorrin‐6y C5‐methyltransferase CbiE
SHALO_1522	SMUL_1558	SMN_1544	SJPD1_1534	cobalt‐precorrin‐6y C15‐methyltransferase [decarboxylating] CbiT
SHALO_1523	SMUL_1559	SMN_1545	SJPD1_1535	cobalt‐precorrin‐2 C20‐methyltransferase CbiL
SHALO_1524	SMUL_1560	SMN_1546	SJPD1_1536	cobalt‐precorrin‐4 C11‐methyltransferase CbiF
SHALO_1525	SMUL_1561	SMN_1547	SJPD1_1537	cobalamin biosynthesis protein CbiG
SHALO_1526	SMUL_1562	SMN_1548	SJPD1_1538	cobalt‐precorrin‐3b C17‐methyltransferase CbiH
SHALO_1527	SMUL_1563	SMN_1549	SJPD1_1539	uroporphyrinogen‐III methyltransferase / Uroporphyrinogen‐III synthase
SHALO_1528	SMUL_1564	SMN_1550	SJPD1_1540	cobalt‐precorrin‐6x reductase CbiJ
SHALO_1529	SMUL_1565	SMN_1551	SJPD1_1541	lipid A export ATP‐binding/permease protein MsbA‐like
SHALO_1530	SMUL_1566	SMN_1552	SJPD1_1542	cobyrinic acid A,C‐diamide synthase CbiA
SHALO_1531	SMUL_1567	SMN_3265	SJPD1_1543	cysteine‐rich CWC protein
SHALO_1532	SMUL_1568	SMN_1553	SJPD1_1544	Precorrin‐2 dehydrogenase (NAD‐dependent)
SHALO_1533			SJPD1_1545	TetR‐like transcriptional regulator
	SMUL_1569	SMN_1554		TetR‐like transcriptional regulator, disrupted by transposase
	SMUL_1570	SMN_1555		ISPsy4, transposition helper protein
	SMUL_1571	SMN_1556		integrase catalytic region
	SMUL_1572	SMN_1557		TetR‐like transcriptional regulator, disrupted by transposase
SHALO_1534	SMUL_1573	SMN_1558	SJPD1_1546	FeS cluster binding motif‐containing flavoprotein
SHALO_1535	SMUL_1574	SMN_1559	SJPD1_1547	Homologue to Rieske proteins, CxH(x)CxxH motif replaced with CxH(x)CxxN
SHALO_1536	SMUL_1575	SMN_1560	SJPD1_1548	putative FMN‐binding protein
SHALO_1537	SMUL_1576	SMN_1561	SJPD1_1549	putative membrane protein

**TABLE A6:** Blastp results of transposase genes detected in the OHR gene region.

Organism	SJPD1_1513: putative transposase			SJPD1_1514: putative transposase		
	Name	aa seq id	Coverage	Name	aa seq id	Coverage
*Sulfurospirillum* sp. JPD‐1	SJPD1_0244	100%	100%	SJPD1_0245	100%	100%
*Sulfurospirillum* sp. JPD‐1	SJPD1_0977			SJPD1_0976		
*Sulfurospirillum* sp. JPD‐1	SJPD1_1067			SJPD1_1066		
*Sulfurospirillum* sp. JPD‐1	SJPD1_1488			SJPD1_1489		
*Sulfurospirillum* sp. JPD‐1	SJPD1_1646			SJPD1_1645		
*Sulfurospirillum multivorans* strain N	SMN_0981			SMN_0980		
*Sulfurospirillum multivorans* strain N	SMN_0280	99%	100%	SMN_0281	100%	100%
*Sulfurospirillum multivorans*	SMUL_0991			SMUL_0990	99%	100%
“*Candidatus* S. diekertiae” SL2‐1	Sdiek1_0868	94%	100%	Sdiek1_0867	95%	100%
“*Candidatus* S. diekertiae” SL2‐2	Sdiek2_0859			Sdiek2_0858	93%	77%
*Sulfurospirillum multivorans*	SMUL_0210	100%	93%	SMUL_0211	100%	100%
*Sulfurospirillum multivorans*	SMUL_0287			SMUL_0288		
*Sulfurospirillum multivorans*	SMUL_1589			SMUL_1588		
*Sulfurospirillum multivorans*	SMUL_2876			SMUL_2877		

Organism	SMUL_1570: ISPsy4, transposition helper protein			SMUL_1571: integrase catalytic region		
	Name	aa seq id	Coverage	Name	aa seq id	Coverage
“*Candidatus* S. diekertiae” SL2‐2	Sdiek2_1043	100%	100%	Sdiek2_1044	100%	100%
*Sulfurospirillum multivorans* strain N	SMN_1555			SMN_1556		
*Sulfurospirillum multivorans*	SMUL_0039	91%	100%	SMUL_0040	91%	100%
*Sulfurospirillum multivorans*	SMUL_0261			SMUL_0260		
*Sulfurospirillum multivorans*	SMUL_0276			SMUL_0275		
*Sulfurospirillum multivorans*	SMUL_1000			SMUL_999		
*Sulfurospirillum multivorans*	SMUL_2742			SMUL_2741		
*Sulfurospirillum halorespirans*	SHALO_2283			SHALO_2284		
“*Candidatus* S. diekertiae” SL2‐1	Sdiek1_1055	99%	83%	Sdiek1_1056	100%	97%
*Sulfurospirillum multivorans*	SMUL_3012	frameshift: not functional		SMUL_3011	98%	97%
*Sulfurospirillum multivorans* strain N	not annotated			SMN_2977		
*Sulfurospirillum multivorans*	SMUL_3014	frameshift: not functional		SMUL_3015	99%	100%
*Sulfurospirillum multivorans* strain N	not annotated			SMN_2979		
*Sulfurospirillum multivorans* strain N	SMN_0037	91%	100%	SMN_0038	90%	97%
*Sulfurospirillum multivorans* strain N	SMN_0992			SMN_0991		
*Sulfurospirillum multivorans* strain N	SMN_0254			SMN_0253	91%	99%
*Sulfurospirillum multivorans* strain N	SMN_0270			SMN_0269		
*Sulfurospirillum multivorans* strain N	SMN_2714			SMN_2713		

Organism	SMN_1525: transposase		
	Name	aa seq id	coverage
*Sulfurospirillum multivorans*	SMUL_0896	100%	100%
*Sulfurospirillum multivorans*	SMUL_1226		
*Sulfurospirillum multivorans*	SMUL_2120		
*Sulfurospirillum multivorans* strain N	SMN_1982		
*Sulfurospirillum multivorans* strain N	SMN_2100		
*Sulfurospirillum multivorans*	SMUL_2437	99%	100%
*Sulfurospirillum multivorans*	SMUL_1516	94%	100%
*Sulfurospirillum halorespirans*	SHALO_2308		
*Sulfurospirillum halorespirans*	SHALO_2374		
*Sulfurospirillum multivorans* strain N	SMN_1501		

**TABLE A7:** Read alignment statistics.

Libraries	Py_Fu_A_ minus_TEX	Py_Fu_A_plus_TEX	Py_Fu_B_ minus_TEX	Py_Fu_B_plus_TEX	Py_PCE_A_minus_TEX	Py_PCE_A_plus_TEX	Py_PCE_B_minus_TEX	Py_PCE_B_plus_TEX
No. of input reads	5469794	6112735	5304494	5014615	5614126	7164779	4375347	7784563
No. of reads—polyA detected and removed	2341132	3371730	1423646	1914606	2339894	3482963	2050260	2828722
No. of reads—single 3’ A removed	515530	477573	327513	501826	590518	675424	421229	1023195
No. of reads—unmodified	2613132	2263432	3553335	2598183	2683714	3006392	1903858	3932646
No. of reads—removed as too short	30904	59388	35863	307333	24179	56722	50310	329515
No. of reads—long enough, used for alignment	5438890	6053347	5268631	4707282	5589947	7108057	4325037	7455048
Total no. of aligned reads	5376025	5997438	5239759	4620634	5538334	7051727	4287282	7323363
Total no. of unaligned reads	62865	55909	28872	86648	51613	56330	37755	131685
Total no. of uniquely aligned reads	3972686	4604113	2281527	3697868	4752217	5418877	3498178	4951861
Total no. of alignments	6815238	7454212	8227512	5580782	6380790	8757868	5136737	9749498
Percentage of aligned reads (compared to no. of input reads)	98.29	98.11	98.78	92.14	98.65	98.42	97.99	94.08
Percentage of aligned reads (compared to no. of long enough reads)	98.84	99.08	99.45	98.16	99.08	99.21	99.13	98.23
Percentage of uniquely aligned reads (in relation to all aligned reads)	73.9	76.77	43.54	80.03	85.81	76.84	81.59	67.62

**TABLE A8:** Mostly regulated genes (Py_PCE vs. Py_Fu), padj: Benjamini–Hochberg‐adjusted p‐value.

Locus_tag	Product	log2FoldChange	*p*adj
SMUL_0188	manganese/zinc/iron chelate uptake transporter (MZT) family, periplasmic‐binding protein	3.706141763	1.29009E‐13
SMUL_0547	heat shock protein Hsp20	3.928639806	4.69236E‐32
SMUL_0914	oleate hydratase	−5.328708478	6.67844E‐66
SMUL_1376	indolepyruvate oxidoreductase subunit IorB	−3.357859244	4.61594E‐13
SMUL_1531	PceA	7.957848413	1.90956E‐93
SMUL_1532	PceB	5.529636956	1.37944E‐10
SMUL_1533	IscU/NifU‐like protein	6.203163354	3.00897E‐36
SMUL_1540	putative membrane protein	4.091494694	9.32673E‐08
SMUL_1541	PceM	6.185575879	6.59202E‐76
SMUL_1542	PceN	7.617250082	9.76877E‐62
SMUL_1543	CbiB	6.784071929	2.80479E‐61
SMUL_1544	threonine phosphate decarboxylase‐like enzyme	7.653246092	4.77859E‐59
SMUL_1545	CobU	7.301835871	2.26884E‐56
SMUL_1546	CbiP	7.229554336	8.21767E‐94
SMUL_1547	CobT	7.450000181	2.15013E‐81
SMUL_1548	cysteine‐rich domain protein	7.15783996	1.56005E‐35
SMUL_1549	CobS	7.110218148	1.42499E‐50
SMUL_1550	CobC	7.570170467	1.10639E‐46
SMUL_1551	CbiK	6.442502207	1.15417E‐79
SMUL_1552	BtuC	6.975106327	8.8242E‐101
SMUL_1553	BtuD	7.322787761	3.64873E‐59
SMUL_1554	BtuF	6.870970537	2.23065E‐63
SMUL_1555	CbiC	6.390476154	2.03371E‐34
SMUL_1556	CbiD	6.293549376	4.05651E‐85
SMUL_1557	CbiE	5.940440235	2.94744E‐41
SMUL_1558	CbiT	6.377525645	1.48587E‐40
SMUL_1559	CbiL	6.826321327	3.45861E‐65
SMUL_1560	CbiF	7.12898108	2.37995E‐69
SMUL_1561	CbiG	6.327298396	5.61016E‐85
SMUL_1562	CbiH	6.783888426	2.94934E‐59
SMUL_1563	uroporphyrinogen‐III methyltransferase / Uroporphyrinogen‐III synthase	5.675055838	1.15417E‐79
SMUL_1564	CbiJ	4.857598806	6.31887E‐71
SMUL_1565	lipid A export ATP‐binding/permease protein MsbA‐like	3.373444956	2.04003E‐15
SMUL_1566	CbiA	4.123923514	6.67467E‐31
SMUL_1567	cysteine‐rich CWC protein	3.537696231	1.02731E‐22
SMUL_1568	SirC	3.517441259	4.30141E‐25
SMUL_1569	TetR‐like transcriptional regulator	3.752933837	2.61372E‐21
SMUL_1573	FeS cluster binding motif‐containing flavoprotein	9.019230255	4.96274E‐44
SMUL_1574	rieske‐like domain‐containing redox protein	8.715025983	3.63129E‐59
SMUL_1575	putative FMN‐binding protein	7.25225219	2.6604E‐107
SMUL_1576	putative membrane protein	7.266423648	1.69691E‐42
SMUL_1679	fumarate hydratase / tartrate dehydratase class I, beta subunit	−3.784971996	1.45393E‐06
SMUL_1680	fumarate hydratase class I / tartrate dehydratase, alpha subunit	−3.631948264	1.66852E‐13
SMUL_1681	anaerobic C4‐dicarboxylate transporter	−3.995272359	1.82261E‐44
SMUL_2666	methyl‐accepting chemotaxis sensory transducer	−4.518658635	5.14879E‐26
SMUL_2667	methyl‐accepting chemotaxis sensory transducer	−4.340873976	2.68256E‐40
SMUL_2817	aspartate ammonia‐lyase	−9.11183091	1.3706E‐150
SMUL_2818	C4‐dicarboxylate transporter DcuA	−8.750538177	4.2635E‐102
SMUL_2819	L‐asparaginase	−8.921077706	5.17731E‐93

**TABLE A9:** OHR genes.

Locus_tag	Product	log2FoldChange	*p*adj
SMUL_1530	alkylhydroperoxidase AhpD family protein	2.288230864	7.52726E‐07
SMUL_1531	PceA	7.957848413	1.90956E‐93
SMUL_1532	PceB	5.529636956	1.37944E‐10
SMUL_1533	IscU/NifU‐like protein	6.203163354	3.00897E‐36
SMUL_1534	two‐component sensor histidine kinase	1.64903125	0.156683843
SMUL_1535	two‐component response regulator	2.183682739	0.001374947
SMUL_1536	RdhA	1.594020272	0.00172506
SMUL_1537	RdhB	0.342863587	0.963904841
SMUL_1538	PceS	1.692624888	1.51154E‐05
SMUL_1539	PceP	0.892719153	0.092756887
SMUL_1540	putative membrane protein	4.091494694	9.32673E‐08
SMUL_1541	PceM	6.185575879	6.59202E‐76
SMUL_1542	PceN	7.617250082	9.76877E‐62
SMUL_1543	CbiB	6.784071929	2.80479E‐61
SMUL_1544	threonine phosphate decarboxylase‐like enzyme	7.653246092	4.77859E‐59
SMUL_1545	CobU	7.301835871	2.26884E‐56
SMUL_1546	CbiP	7.229554336	8.21767E‐94
SMUL_1547	CobT	7.450000181	2.15013E‐81
SMUL_1548	cysteine‐rich domain protein	7.15783996	1.56005E‐35
SMUL_1549	CobS	7.110218148	1.42499E‐50
SMUL_1550	CobC	7.570170467	1.10639E‐46
SMUL_1551	CbiK	6.442502207	1.15417E‐79
SMUL_1552	BtuC	6.975106327	8.8242E‐101
SMUL_1553	BtuD	7.322787761	3.64873E‐59
SMUL_1554	BtuF	6.870970537	2.23065E‐63
SMUL_1555	CbiC	6.390476154	2.03371E‐34
SMUL_1556	CbiD	6.293549376	4.05651E‐85
SMUL_1557	CbiE	5.940440235	2.94744E‐41
SMUL_1558	CbiT	6.377525645	1.48587E‐40
SMUL_1559	CbiL	6.826321327	3.45861E‐65
SMUL_1560	CbiF	7.12898108	2.37995E‐69
SMUL_1561	CbiG	6.327298396	5.61016E‐85
SMUL_1562	CbiH	6.783888426	2.94934E‐59
SMUL_1563	CobA/HemD	5.675055838	1.15417E‐79
SMUL_1564	CbiJ	4.857598806	6.31887E‐71
SMUL_1565	lipid A export ATP‐binding/permease protein MsbA‐like	3.373444956	2.04003E‐15
SMUL_1566	CbiA	4.123923514	6.67467E‐31
SMUL_1567	cysteine‐rich CWC protein	3.537696231	1.02731E‐22
SMUL_1568	SirC	3.517441259	4.30141E‐25
SMUL_1569	TetR‐like transcriptional regulator	3.752933837	2.61372E‐21
SMUL_1570	ISPsy4, transposition helper protein	−0.255044852	0.963904841
SMUL_1571	integrase catalytic region	−0.08886508	0.986159508
SMUL_1572	TetR‐like transcriptional regulator	0.766545409	0.854250437
SMUL_1573	FeS cluster binding motif‐containing flavoprotein	9.019230255	4.96274E‐44
SMUL_1574	rieske‐like domain‐containing redox protein	8.715025983	3.63129E‐59
SMUL_1575	putative FMN‐binding protein	7.25225219	2.6604E‐107
SMUL_1576	putative membrane protein	7.266423648	1.69691E‐42

**TABLE A10:** Identified transcriptional units in the OHR gene region, locations of the transcriptional start sides (TSSs) in the genome of *S. multivorans*, and the genes organized in these operons.

Locus_tag	TSS_location	Genes
pSMUL_1530	1490384	SMUL_1530
p*pceAB*	1491183	SMUL_1531‐1532
pSMUL_1533	1493874	SMUL_1533
pTCS1	1496519	SMUL_1534‐1535
pTCS2	1501090	SMUL_1538‐1539
pSMUL_1540	1501260	SMUL_1540
p*pceMN*/B12	1531093	SMUL_1541‐1569
pSMUL_1573	1528995	SMUL_1573‐1576

**TABLE A11:** Quantification of the transcript level at the end of the seventh transcriptional unit of the OHR gene region in *S*. *halorespirans* by the use of RT‐qPCR. C(t) values of three intergenic regions are given. The first intergenic region is located between SHALO_1504/1505 and serves as a negative control, the second region is located between SHALO_1532/1533, and the third region is located between SHALO_1533/1534. Genomic DNA (gDNA) of *S. halorespirans* was used as a positive control. Water served as an internal control. Complementary (cDNA) was used for the RT‐ control.

Intergenic region content	Primer	Template	C(t) Mean	C(t) Std. Dev
Sample: SHALO_1504 ‐> SHALO_1505	T774/T775	Sh_cDNA	N/A	N/A
RT‐ Ctrl: SHALO_1504 ‐> SHALO_1505	T774/T775	Sh_cDNA	N/A	N/A
Pos Ctrl: SHALO_1504 ‐> SHALO_1505	T774/T775	Sh_gDNA	23.83	1.627
Neg Ctrl: SHALO_1504 ‐> SHALO_1505	T774/T775	H_2_O	N/A	N/A
Sample: SHALO_1532 ‐> SHALO_1533	T776/T777	Sh_cDNA	22.48	0.657
RT‐ Ctrl: SHALO_1532 ‐> SHALO_1533	T776/T777	Sh_cDNA	N/A	N/A
Pos Ctrl: SHALO_1532 ‐> SHALO_1533	T776/T777	Sh_gDNA	16.65	0.248
Neg Ctrl: SHALO_1532 ‐> SHALO_1533	T776/T777	H_2_O	N/A	N/A
Sample: SHALO_1533 ‐> SHALO_1534	T778/T779	Sh_cDNA	29.94	0.445
RT‐ Ctrl: SHALO_1533 ‐> SHALO_1534	T778/T779	Sh_cDNA	N/A	N/A
Pos Ctrl: SHALO_1533 ‐> SHALO_1534	T778/T779	Sh_gDNA	15.77	0.351
Neg Ctrl: SHALO_1533 ‐> SHALO_1534	T778/T779	H_2_O	N/A	N/A
